# The phosphoglycerate kinase 1 variants found in carcinoma cells display different catalytic activity and conformational stability compared to the native enzyme

**DOI:** 10.1371/journal.pone.0199191

**Published:** 2018-07-11

**Authors:** Annarita Fiorillo, Maria Petrosino, Andrea Ilari, Alessandra Pasquo, Alessandra Cipollone, Maristella Maggi, Roberta Chiaraluce, Valerio Consalvi

**Affiliations:** 1 Department of Biochemical Sciences “A. Rossi Fanelli”, Sapienza University of Rome, Rome, Italy; 2 CNR-Institute of Molecular Biology and Pathology, Rome, Italy; 3 ENEA CR Frascati, Diagnostics and Metrology Laboratory, FSN-TECFIS-DIM, Frascati, Italy; 4 Department of Molecular Medicine, Unit of Immunology and General Pathology, University of Pavia, Pavia, Italy; Universidad de Granada, SPAIN

## Abstract

Cancer cells are able to survive in difficult conditions, reprogramming their metabolism according to their requirements. Under hypoxic conditions they shift from oxidative phosphorylation to aerobic glycolysis, a behavior known as Warburg effect. In the last years, glycolytic enzymes have been identified as potential targets for alternative anticancer therapies. Recently, phosphoglycerate kinase 1 (PGK1), an ubiquitous enzyme expressed in all somatic cells that catalyzes the seventh step of glycolysis which consists of the reversible phosphotransfer reaction from 1,3-bisphosphoglycerate to ADP, has been discovered to be overexpressed in many cancer types. Moreover, several somatic variants of PGK1 have been identified in tumors. In this study we analyzed the effect of the single nucleotide variants found in cancer tissues on the PGK1 structure and function. Our results clearly show that the variants display a decreased catalytic efficiency and/or thermodynamic stability and an altered local tertiary structure, as shown by the solved X-ray structures. The changes in the catalytic properties and in the stability of the PGK1 variants, mainly due to the local changes evidenced by the X-ray structures, suggest also changes in the functional role of PGK to support the biosynthetic need of the growing and proliferating tumour cells.

## Introduction

Carcinomas are the most common type of cancer. According to the American Cancer Society, in 2016 the overall estimate is of 1685210 new cases of cancer worldwide and among them 61000 cases of female breast carcinoma in situ are expected [1]. Cancer cells are able to adapt to survive in difficult conditions, like for example in O_2_ deficiency, through a reprogramming of their metabolic machinery according to their requirements [2]. Among these, the hallmark of the metabolic reprogramming is the shift from oxidative phosphorylation to aerobic glycolysis that allows tumor cells to survive under hypoxic conditions [3], an adaptative behaviour described nearly 100 years ago and known as the Warburg effect. In the last years, glycolytic enzymes, and their role in cancer metabolism, have been the object of several studies and they have been identified as potential targets for alternative anticancer therapies [4, 5].

A central enzyme in glycolysis is the phosphoglycerate kinase 1 (PGK1). This ubiquitous glycolytic enzyme is expressed in all somatic cells, where it provides energy in form of ATP through the reversible phosphotransfer reaction from 1,3-bisphosphoglycerate (1,3-BPG) to MgADP in order to produce 3-phosphoglycerate (3-PG) and MgATP in the presence of free magnesium [6,7]. Deficiency of PGK1 has been associated with hereditary non-spherocytic haemolytic anaemia (HNSHA), a rare disorder that is caused by a variety of inherited defects in glycolysis [8].

**Fig 1:**
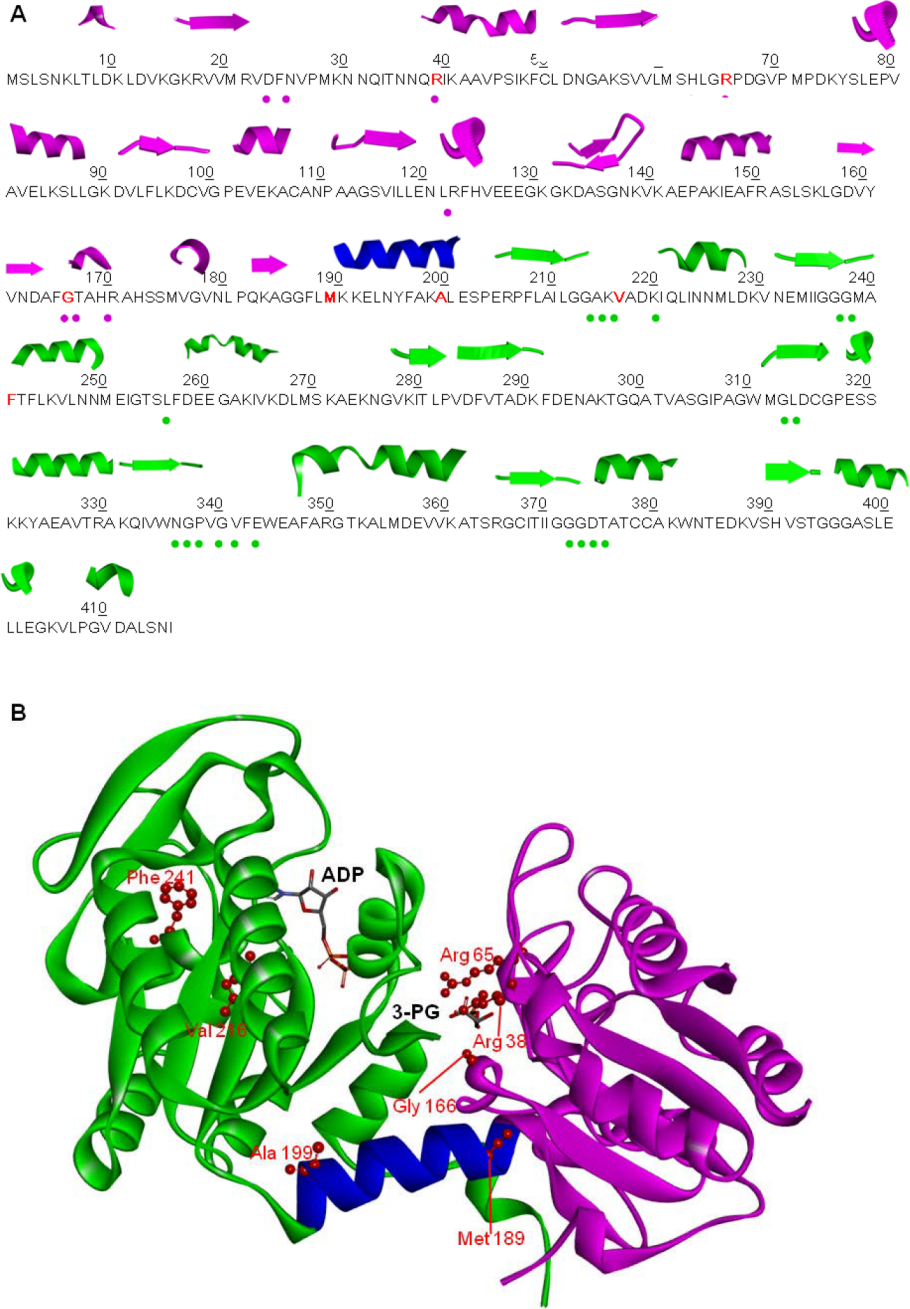
Amino acid sequence and structure of PGK1. N-terminal domain (violet), C-terminal domain (green), hinge region (blue) (PDB: 2XE7, open conformation). (A) Secondary structural elements are shown at the top of the amino acid sequence. Mutated residues are depicted in bold red. The dots under the sequence represent the residues involved in 3-PG or 1,3-BPG binding (pink dot) and in ATP or ADP binding (green dot). (B) Location of the mutations on PGK1 structure (open conformation). Mutated residues are depicted in scaled ball and stick and 3-PG and ADP in stick.

The structure of human PGK1 was solved in the open conformation (PDB code: 2XE7) [7] in the partially closed conformation (PDB code: 2ZGV) [9] and in closed conformation bound to a transition state analogue (PDB code: 2WZB) [10] as well as in complex with inhibitors (PDB codes: 4O33, 4O3F) [11]. PGK1 is a monomeric enzyme of 417 amino acids formed by two distinct α-helical domains of equal size, the N- and C-terminal domains connected by a hinge region (Fig 1). The N-terminal domain binds 3-PG or 1,3-BPG while the C-terminal domain binds MgADP or MgATP. During the catalytic cycle four hinge points mediate interdomain motions that result in the flexible region bending, allowing the two domains to approach each other bringing the catalytic residues in the right position. During this transition phase, a large number of conformational rearrangements, triggered by the simultaneous binding of the two substrates, lead the enzyme from its open form, which has the highest affinity for the substrates, to its closed form in which the enzyme performs the transfer of the phosphoryl group fulfilling its catalytic activity [6].

Interestingly, similarly to many other glycolytic enzymes, PGK1, in addition to its metabolic function, may acquire different functions. This enzyme may be secreted in the extracellular environment by tumour cells and act as a thiol reductase regulating angiogenesis [12]. In addition, translocation of PGK1 to the nucleus is related to binding to alpha DNA polymerase [13]. Notably, under hypoxic conditions, PGK1 may translocate from cytoplasm to mitochondrion where it may act as a protein kinase and phosphorylate different protein substrates [14]. Interestingly, the protein kinase activity of PGK1 has been related to initiation of autophagy [15, 16].

PGK1 is regulated by hypoxia-induced factor-1ɑ (HIF-1ɑ), the most important factor involved in the cellular response to hypoxia [12]. Several solid tumors like prostate cancer, breast cancer, pancreatic ductal adenocarcinoma, multidrug-resistant ovarian cancer and metastatic gastric cancer exhibit an increased expression of glycolytic enzymes such as PGK1 to generate ATP in hypoxic conditions [17–21]. The elevated levels of PGK1 protein, detected in the serum of patients affected by pancreatic cancer [22, 23] and in breast cancer tissues [23], suggest a plausible use of PGK1 as a cancer biomarker. Indeed, despite all the observations that report PGK1 overexpression in many cancer types, the role of this enzyme in tumorigenesis is yet unclear [14].

In different cancer types, somatic mutations of PGK1 have been identified, as reported in COSMIC (Catalogue of Somatic Mutations in Cancer) (http://cancer.sanger.ac.uk/cosmic), a database which collects somatic mutation identified in human cancers [24]. Most of these variants are missense ones, also known as nonsynonymous single nucleotide variants (nsSNVs), occurring in the coding region and leading to a polypeptide sequence with amino acid substitutions [25]. By controlling ATP and 3-PG levels, PGK1 plays an important role in coordinating energy production with biosynthesis and redox balance, so mutations of this enzyme can be responsible for alterations of metabolic profile in different cancer cell lines.

The effect of nsSNVs on protein stability, protein-protein interactions and protein functions has been investigated for several other protein families [26–29]. Indeed, large-scale computational studies utilizing structural information indicate that a single amino acid substitution will affect either protein-protein interactions and protein stability [30, 31]. Analysis of physico-chemical properties of natural variants may be helpful to reveal local structural changes that may not affect the overall folding of the structure [28]. Thus, only a detailed experimental analysis can unequivocally reveal the effect of the missense mutations on protein function and/or stability [32].

In this study we selected seven PGK1 nsSNVs found in cancer tissues and annotated in the COSMIC database. The mutated residues are located in the 3-PG binding site, in the flexible hinge region that allows the two domains of the enzyme to approach during the catalytic cycle, and in the MgADP binding site in the C-terminal domain (Fig 1). Herewith we report the effects of nsSNV on PGK1 by analysing the impact of single amino acid replacements on the variants structure, on their conformational stability, and on their catalytic activity compared to the wild type protein.

## Materials and methods

### Plasmids and site-directed mutagenesis

PGK1 wild type plasmid was kindly provided by Dr. Maristella Maggi, University of Pavia [PMID: 22348148]. Quick Change Site-Directed Mutagenesis Kit (Stratagene) was used to introduce single mutations in the gene encoding for the wild type PGK1 and inserted into the plasmid backbone. The mutagenic oligonucleotides used to obtain the seven selected variants (i.e, R38M, R65W, G166D, M189I, A199V, V216F, and F241S) are listed in S1 Table.

The presence of the desired mutations and the absence of unwanted additional mutations were confirmed by plasmid sequencing of the insert.

### Protein expression and purification

Recombinant PGK1 protein has been expressed and purified as described in [33] with minor modifications. PGK1 wild type and mutants were expressed in *E*. *coli* strain BL21(DE3). 15 mL of overnight culture containing 100 μg/mL ampicillin were used to inoculate 500 mL LB cultures containing ampicillin as antibiotic at a final concentration of 50 μg/mL. Cultures were grown at 37°C until optical density at 600 nm (OD_600_) reached 0.6, at which point the protein expression was induced by adding 0.5 mM isopropyl-β-D-thiogalactoside (Sigma-Aldrich). Induced cultures were grown at 37°C for 5 h in vigorous shaking. At the end of induction, cells were collected by centrifugation and the pellet frozen.

To purify the enzymes, cells were resuspended in 45 mL of Binding buffer (20 mM Tris-HCl pH 8.0, 1 mM EDTA, 1 mM tris(2-carboxyethyl)phosphine (TCEP)) containing a cocktail of EDTA-free protease inhibitors (Sigma-Aldrich) and disrupted by sonication in a Vibracell sonicator with 5 s boosts and 9 s pause, on ice. The lysate was cleared by centrifugation at 15000 rpm and the supernatant, after an additional centrifugation, was applied to a DEAE-Sepharose FF column (GE Healthcare) previously equilibrated in Binding buffer, to remove endogenous protein contaminants and nucleic acids. In the above described conditions, the recombinant PGK1, eluted in the unbound fraction, was concentrated to 2 mL using an Amicon concentrator Ultra-15 (Millipore). In order to remove protein aggregates, concentrated protein was loaded onto a Superdex 200 300/10 gel filtration column equilibrated in 20 mM Tris-HCl pH 8.0, 1 mM EDTA, 200 mM NaCl, 2 mM DTT using an AKTA FPLC system (GE Healthcare). The protein was eluted by isocratic flow at 1.0 mL/min. 2 mL fractions were collected and purity and molecular weight of the protein were checked by SDS–PAGE on a pre-casted NuPage 4–12% bis-Tris polyacrylamide gel (Invitrogen). Gels were stained with Coomassie blue R-250. Protein concentration was determined spectrophotometrically using a molar absorptivity coefficient (ε_280_) corresponding to 33460 M^-1^cm^-1^ for the variant R65W and 28335 M^-1^cm^-1^ for wild type and the other variants, based on a molecular mass of 44.615 kDa, and calculated according to Gill and Hippel [34]. Pure protein was used for all structural and stability experiments. The enzyme obtained from 1 liter culture was approximately 65 mg in the case of wild type. High level of expression, ranging from 50 mg (V216F and F241S) to 87 mg (R38M), was obtained for all the mutant enzymes too.

### Spectroscopic measurements

Intrinsic fluorescence emission measurements were recorded at a protein concentration of 110 μg/mL for the R65W variant and 130 μg/mL for wild type and all the other variants (0.08 AU_280nm_), in 20 mM Tris-HCl pH 8.0, 200 mM NaCl and 0.2 mM DTT, in a LS50B spectrofluorimeter (Perkin-Elmer) using a 1.0 cm path length quartz cuvette. Intrinsic fluorescence emission spectra were recorded from 300 to 450 nm (1 nm sampling interval), with the excitation wavelength set at 295 nm. Far-UV (190–250 nm) CD spectra were monitored at 20°C at a protein concentration ranging over 130–170 μg/mL, using a 0.1 cm path length quartz cuvette, in 20 mM Tris-HCl pH 8.0, 200 mM NaCl and 0.2 mM DTT. Near-UV (250–320 nm) CD spectra were recorded in a 1.0 cm path length quartz cuvette at a protein concentration ranging between 1.50 and 1.70 mg/mL, in 20 mM Tris-HCl pH 8.0, 200 mM NaCl, 1.0 mM EDTA and 2.0 mM DTT. CD measurements were carried out in a JASCO-815 spectropolarimeter (Jasco, Easton, MD, USA) and the results are expressed as the mean residue ellipticity ([Θ]), assuming a mean residue molecular mass of 110 per amino acid residue. All spectroscopic measurements were carried out at 20°C.

### Enzyme activity assay and kinetic studies

PGK1 activity was determined at 20°C, with 3-PG and MgATP as substrates, by glyceraldehyde-3-phosphate dehydrogenase (GAPDH) coupled spectrophotometric assay according to [33]. The standard reaction mixture contained 100 mM Tris-HCl pH 8.0, 0.5 mM EDTA, 2 mM MgCl_2_, 0.24 mM NADH, 4U/0.5mL GAPDH, 15.0 mM 3-PG, and 5.0 mM MgATP, in a final volume of 0.5 mL. Kinetic parameters for PGK1 activity towards 3-PG were determined at 20°C by using at least 10 different concentrations of 3-PG under conditions identical to those described above, at 5.0 mM MgATP fixed substrate concentration. The reaction was started by adding different amounts of PGK1 ranging from 4 ng to 20 μg. All measurements were performed in triplicate in a Lambda 16 computerized spectrophotometer (Perkin-Elmer). Kinetic data were analysed according to [33], using GraphPadPrism 5.04. Results are reported as the mean of three experiments from different protein preparations.

### Temperature dependence of PGK1 activity

The activity assay mixture, containing 100 mM Tris-HCl pH 8.0, 0.5 mM EDTA, 2 mM MgCl_2_, 0.24 mM NADH, 4U/0.5mL GAPDH, 5.0 mM MgATP and 0.2 mM 3-PG in 0.5 mL final volume, was incubated at increasing temperature in a thermostated cuvette. 4 μL of pure enzyme, at 10°C, were added to 0.5 mL of the assay mixture equilibrated at the desired temperature to start the reaction. The final enzyme concentration was 0.18–12.0 nM. The solution was mixed in the thermostated cuvette and the absorbance at 340 nm was continuously monitored for 10 min. The changes of enzyme activity as a function of temperature was fitted nonlinearly to the Arrhenius equation using GraphPadPrism 5.04 to obtain the activation energies (*E*_a_) for the catalytic reaction

$$k=Ae^{-Ea/RT}$$ (1)

where *k* (s^-1^) is the rate constant at temperature T (K), *A* is a reaction specific quantity, *R* the gas constant (1.987 cal x mol^-1^ x K^-1^) and *E*_a_ the activation energy of the reaction, as described in [27].

### Urea-induced unfolding equilibrium

PGK1 wild type and variants (110–160 μg/mL final concentration) were incubated at 20°C at increasing concentrations of urea (0−8 M) in 20 mM Tris-HCl pH 8.0, 200 mM NaCl, 5.0 mM MgCl_2_ and 0.2 mM DTT. After 10 min, a time sufficient to reach equilibrium, intrinsic fluorescence emission and far-UV CD spectra (0.2-cm cuvette) were recorded in parallel at 20°C. To test the reversibility of the unfolding, PGK1 wild type and variants were unfolded at 20°C in 7.5 M urea at protein concentration ranging over 1.10–1.60 mg/mL in 20 mM Tris-HCl, pH 8.0, in the presence of 2 mM DTT, 5 mM MgCl_2_ and 200 mM NaCl. After 10 min, refolding was started by 10-fold dilution of the unfolding mixture at 20°C into solutions of the same buffer used for unfolding containing decreasing urea concentrations. The final protein concentration was 110–160 μg/mL. After 2 h, intrinsic fluorescence emission and far-UV CD spectra were recorded at 20°C. All denaturation experiments were performed in triplicate.

### Thermal stability

PGK1 wild type and variants (110–160 μg/mL) were heated from 20°C to 80°C in 20 mM Tris-HCl, pH 8.0, 200 mM NaCl, 0.2 mM DTT, in a 0.1 cm quartz cuvette with a heating rate of 1 degree x min^-1^ controlled by a Jasco programmable Peltier element as described in [27, 29]. The dichroic activity at 222 nm and the photomultiplier were continuously monitored in parallel every 0.5°C [35]. The solvent contribution at the various temperatures was taken into consideration for all thermal scans. Melting temperature (T_m_) values were calculated by taking the first derivative of the ellipticity at 222 nm with respect to temperature, as described in [29]. All denaturation experiments were performed in triplicate.

### Data analysis

Far-UV CD spectra recorded as a function of urea concentration were analyzed by a singular value decomposition algorithm (SVD) using the software MATLAB (Math-Works, South Natick, MA) to remove the high frequency noise and the low frequency random errors and determine the number of independent components in any given set of spectra, as described in [27].

The changes in intrinsic fluorescence emission spectra at increasing urea concentrations were quantified as the intensity-averaged emission wavelength, $\left(\bar{\lambda }\right)$, [36] calculated according to

$$\bar{\lambda }=\Sigma (I_{i}\lambda_{i})/\Sigma \left(I_{i}\right)$$ (2)

where λ_i_ and *I*_i_ are the emission wavelength and its corresponding fluorescence intensity at that wavelength, respectively. This quantity is an integral measurement, negligibly influenced by the noise, which reflects changes in the shape and position of the emission spectrum.

Urea-induced equilibrium unfolding transitions monitored by far-UV CD ellipticity and intrinsic fluorescence emission changes were analysed by fitting baseline and transition region data to a two-state linear extrapolation model [37] according to

$$\Delta G_{\mathrm{unfolding}}=\Delta G^{H_{2}O}+m\left[Urea\right]=-RTlnK_{\mathrm{unfolding}}$$ (3)

where Δ*G*_unfolding_ is the free energy change for unfolding for a given denaturant concentration, $\Delta G^{H_{2}O}$ the free energy change for unfolding in the absence of denaturant and *m* a slope term which quantifies the change in Δ*G*_unfolding_ per unit concentration of denaturant, *R* the gas constant, T the temperature and *K*_unfolding_ the equilibrium constant for unfolding. The model expresses the signal as a function of denaturant concentration:

$$y_{i}=\frac{y_{N}+s_{N}{\left[X\right]}_{i}+\left(y_{U}+s_{U}{\left[X\right]}_{i}\right)*\exp\left[\left(-\Delta G^{H_{2}O}-m{\left[X\right]}_{i}\right)/RT\right]}{1+\exp\left[\frac{\left(-\Delta G^{H_{2}O}-m{\left[X\right]}_{i}\right)}{RT}\right]}$$ (4)

where *y*_i_ is the observed signal, *y*_U_ and y_N_ are the baseline intercepts for unfolded and native protein, *s*_U_ and *s*_N_ are the baseline slopes for the unfolded and native protein, [X]_i_ the denaturant concentration after the ith addition, $\Delta G^{H_{2}O}$ the extrapolated free energy of unfolding in the absence of denaturant, *m* the slope of a Δ*G*_unfolding_ versus [X] plot. The denaturant concentration at the midpoint of the transition, [Urea]_0.5_, according to Eq (3), is calculated as:

$${\left[Urea\right]}_{0.5}=\Delta G^{H_{2}O}/m_{}$$ (5)

All unfolding transition data were fitted by using Graphpad Prism 5.04.

### Variants crystallization, data collection, structures solution

The protein samples used for crystallization trials contained PGK1 variants at a concentration of 12–18 mg/mL in Tris-HCl 20 mM pH 7.0, in the presence of 10 mM ADP, 25mM MgCl_2_, 50 mM 3-PG and 10 mM DTT. Crystals were obtained at 298 K by the hanging-drop vapour diffusion method, sealing symmetric drops (1.0+1.0 μL) over 500 μL of reservoir solution.

We performed crystallization experiments with the PGK1 variants V216F, G166D, M189I, R38M in order to obtain the structure of the protein-ligands complexes in both closed and partially closed conformation. The detailed crystallization conditions for all the structures solved are indicated in S2 Table. First we tested the conditions reported in the literature for the partially closed conformation of wild type PGK1 [9] based on Na/K phosphate, obtaining diffracting crystals (0.1 × 0.2 × 0.3 mm^3^) for V216F and M189I. Then a high throughput crystallization screening (HTS) approach was employed for G166D and R38M variants. The crystallization robot (Crystal Phoenix, Art Robbins Instruments) available at the bio-crystal facility at the IBPM-CNR (c/o Department of Biochemical Sciences, Sapienza University of Rome) allowed us to set up up-to crystallization trials using commercial crystallization kits (Crystal Screens I, II, Wizard I, II and Index HT, Hampton Research). The best crystals of R38M and G166D mutants have been obtained in sodium citrate. We also tried to crystallize the closed form of the mutants by adding 20 mM NH_4_F in the protein solutions defined above in order to mimic the transition state of the enzyme. Again we first tested the conditions already reported for the wild type PGK1 [10], obtaining crystals only for the M189I mutant. Then we performed HTS with the other mutants without success.

A single-wavelength data set was collected at 100 K from each crystal and processed with XDS [38] and Aimless [39]. The structures were determined by molecular replacement with the program MOLREP (CCP4 suite) [40] using the PDB entries 2WZB and 2ZGV as search models for closed and partially closed conformations respectively. Refinement has been performed using the maximum-likelihood method with the program REFMAC [41] and model building has been done by using Coot [42]. Crystal parameters, beamlines specifications, data collection parameters and refinement statistics are reported in S2 Table.

## Results

In this study we selected seven PGK1 variants (R38M, R65W, G166D, M189I, A199V, V216F and F241S) mined from the COSMIC database (http://cancer.sanger.ac.uk/cosmic) [24] and associated to human carcinoma. Most of these PGK1 variants, M189I, A199V, V216F and F241S (Fig 1), are reported to be found in breast carcinoma; the other variants, R38M, R65W and G166D are reported to be present in lung, liver and endometrium carcinoma. The location of the selected mutants mapped onto the PGK1 structure is shown in Fig 1. Residues R38, R65 and G166 are located in the enzyme 3-PG binding site (Fig 1) at the N-terminal domain; in particular, the residues R38 and G166 are part of two helices, whereas the residue R65 is located in a loop (Fig 1). R38 and R65 are involved in 3-PG binding; in particular, R65 NH1 and NH2 groups are involved in an electrostatic interaction with the carboxyl group of 3-PG, whereas R65 Nε and NH2 groups acquire contacts with the phosphate group of 3-PG [43]. G166 is located in the 3-PG binding pocket interacting with the substrate C2 through its main carboxylic group. M189 and A199 are located at the edges of the helix that represents the flexible hinge region that allow the two domains of the enzyme to approach each other during the catalytic cycle (Fig 1). Residue V216 is located in a turn, in proximity of the nucleotide-binding A214 residue; F241 is part of a helix, and they both belong to the MgADP binding pocket, located at the protein C-terminal domain (Fig 1). Among the mutations studied, only R65W involves a surface exposed residue, all others variants, R38M, G166D, M189I, A199V, V216F and F241S are more buried. We generated recombinant protein for each of the identified mutants using site directed mutagenesis and available bacterial expression systems. Introduction of these mutations resulted in soluble recombinant proteins and allowed us to study the consequences of the mutations on PGK1 thermal and thermodynamic stability and the kinetic activity.

### Spectroscopic characterization of PGK1 wild type and variants

The conformation in solution of all PGK1 variants was studied by CD and fluorescence spectroscopy. The near-UV CD spectrum of PGK1 wild type represents the spectral contributions of all aromatic residues and is characterized by a strong positive peak centred at around 292 nm, flanked by a shoulder at 286 nm and by negative contributions in the region between 255 and 270 nm, with two peaks centred at 262 and 268 nm (S1 Fig). R38M, G166D, A199V and V216F display near-UV CD spectra closely similar to that of the wild type, except for a slight difference at 277 nm observed for G166D (S1 Fig). Significant differences are observed in the near-UV CD spectra of M189I, F241S and R65W (S1 Fig). In the case of M189I and F241S, the contributions at 262 nm and 268 nm are positive; in addition, the spectrum of M189I loses fine structure features in the region between 270 and 280 nm. The variant R65W shows a near-UV CD spectrum that significantly differs from that of the wild type in intensity, consistent with the substitution of an arginine residue with a tryptophan (S1 Fig). The fluorescence spectra of wild type and variants are centred at the same maximum emission wavelength at around 350 nm, characteristic of tryptophan contribution, with differences in the relative fluorescence emission intensity which is significantly decreased for A199V and increased for R65W, M189I and V216F (S1 Fig).

The far-UV CD spectra of PGK1 wild type and variants show the typical local minimum contributions of alpha helical proteins at around 208 nm and at 222 nm (S1 Fig). The molar ellipticity ratio at 222 and at 208 nm (Θ_222_/Θ_208_) is indicative of interhelical contacts present in helix bundle and coiled coil structures and it is generally used to distinguish between coiled coil helices (≥1.0) and non-interacting helices (0.8–0.9) [44,45]. The Θ_222_/Θ_208_ is 1.2 for the wild type, it is decreased to 1.1 only for the variants R38M and M189I and it is increased to 1.3 for V216F (S1 Fig). Notably, in the case of M189I the far-UV dichroic activity is decreased with respect to that of the wild type (S1 Fig). These differences in secondary structure may suggest differences in interhelical interactions and dynamic fluctuation in solution for some of PGK1 variants.

### PGK1 wild type and variants: Kinetic properties and temperature dependence of enzyme activity

#### Enzymatic assays of PGK1 wild type and variants

Kinetic properties of PGK1 wild type and variants were investigated by monitoring the reverse reaction at 20°C at a fixed concentration of free Mg^2+^ and using 3-PG as variable substrate [33]. All the variants display altered kinetic properties, in comparison with the wild type. The kinetic properties of wild type, M189I and F241S, revealed a non Michaelis–Menten behaviour, as indicated by double reciprocal plots typical of substrate activation (data not shown). PGK1 specific activity, analysed using 3-PG as variable substrate over the concentration range 0.05–15.0 mM, is decreased for all the variants with the exception of F241S that displays a two fold increase of activity (Table 1). Notably, all the kinetic parameters indicate a significant decrease of the catalytic efficiency (*k*_cat_/*K*_M_), particularly evident for R38M which displays a catalytic efficiency 10 milion fold lower than that of the wild type. All the variants display an increase of 3-PG *K*_M_ values, particularly evident for F241S that shows a *K*_M_ value 14-fold higher than that of the wild type (Table 1).

**Table 1:** Kinetic parameters of PGK1 wild type and variants.

	[PGK1] (nM)	[3PG] (mM)	*K*_M_ (mM)	*k*cat (s-1)	*k*cat/ *K*_M_ (s-1 mM-1)	Vmax (μM/min)	Specific activity (μM/min•μg)
Wild type	0.18	0.05–15.0	0.40 ± 0.03	89.8 ± 1.5	224.5	0.97	248.7
R38M	9.04 x10^5^	3.00–15.0	3.15 ± 0.40	(7.2 ± 0.5) x10^-6^	2.28x10^-6^	0.39	0.02
R65W	0.35	1.00–15.0	1.61 ± 0.13	23.7 ± 0.5	14.7	0.50	63.8
G166D	0.70	0.90–15.0	1.54 ± 0.15	5.9 ± 0.2	3.8	0.25	15.9
M189I	0.18	0.20–15.0	0.58 ± 0.50	30.5 ± 1.4	52.7	0.33	84.6
A199V	0.18	0.80–15.0	1.95 ± 0.15	76.6 ± 1.7	39.3	0.83	212.0
V216F	7.00	0.10–15.0	0.78 ± 0.05	1.9 ± 0.03	2.4	0.93	5.5
F241S	0.18	0.20–15.0	5.87 ± 0.7	213.0 ± 14.0	31.5	2.30	515.4

PGK1 activity was determined at 20°C, with 3-PG and 5 mM MgATP (fixed substrate concentration), as described in Materials and Methods. Kinetic parameters for PGK1 activity were determined at 20°C by using at least at 10 different concentrations of 3-PG. Data are reported as the mean ± SE of the fit.

#### Temperature dependence of enzyme activity

**Fig 2:**
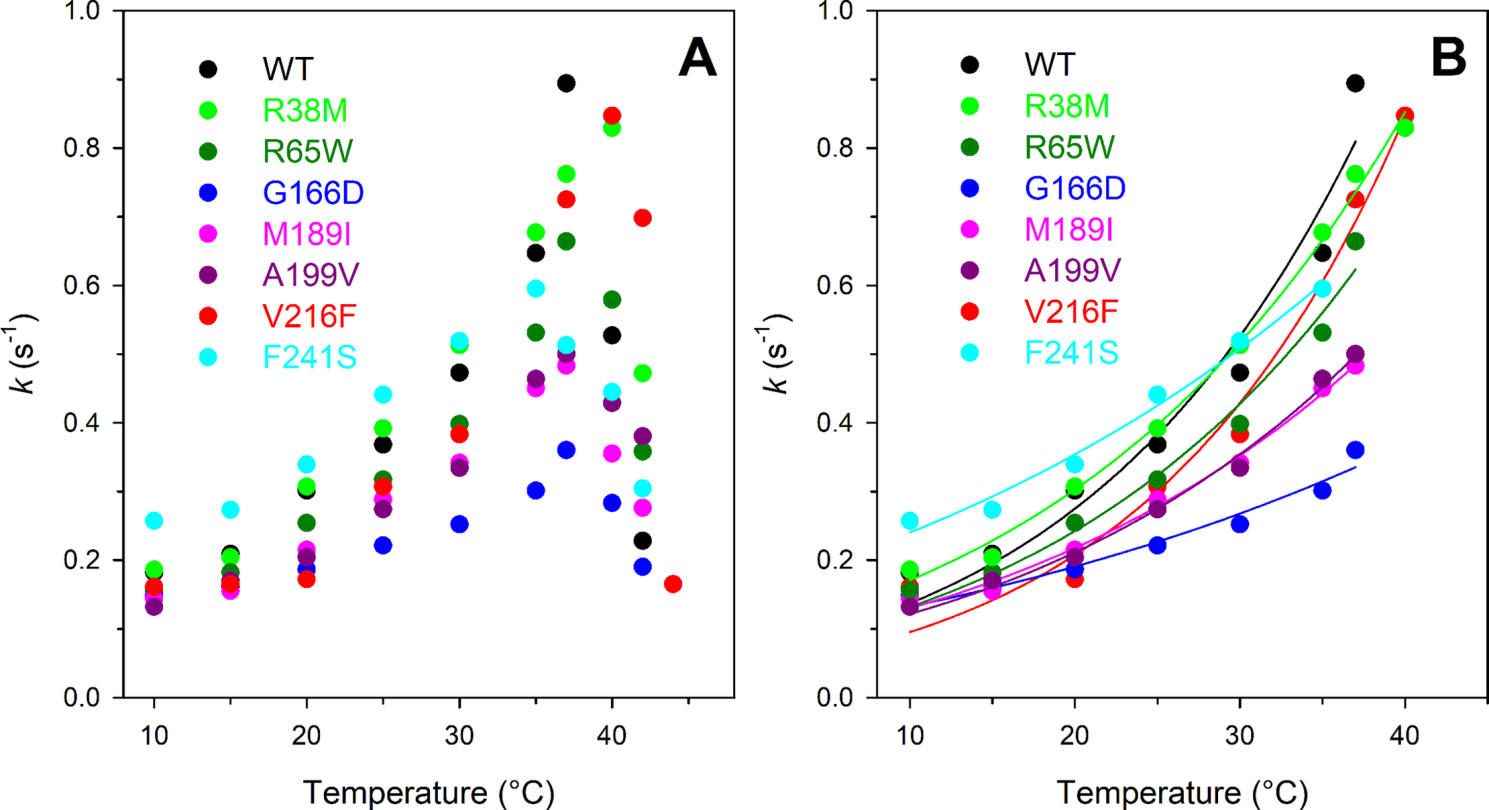
Effect of temperature on kinase activity of PGK1 wild type and variants. (A) Temperature dependence of kinase activity of PGK1 wild type and variants. (B) Non-linear fit of the temperature dependence of PGK1 activity to the Arrhenius equation Eq (1). Assays were performed under the conditions described in Materials and Methods, using 0.18–12.0 nM enzyme.

The kinase activity of PGK1 wild type and variants was analysed as a function of temperature (10–42°C) using a low concentration of 3- PG substrate (0.02 mM, well below the *K*_M_ value) (Fig 2) to study the enzymatic release of substrate and product that may be related to its flexibility. The optimal temperatures for catalysis, in these conditions, were estimated to be 37°C for the wild type and most of the variants, around 35°C for F241S, and around 40°C for R38M and V216F (Table 2 and Fig 2). As shown in Table 2, the lowest activation energy value (*E*_a_) is that of the F241S variant (6.39 kcal/mol) which is also the variant displaying a *k*_cat_ higher than that of the wild type enzyme (213.0 vs 89.8 s^-1^). As mentioned before, the activation energies have been measured at a 3-PG concentration of 0.02 mM, well below the *K*_M_ value. At this substrate concentration, the enzyme is not saturated, the complex enzyme-3-PG-MgATP may dissociate to form again the enzyme-MgATP complex and free 3-PG or may catalyse the formation of the two products MgADP and 1,3-BPG. The activation energy measured in these conditions reflects the enzymatic release of both the 3-PG substrate and the products and it may be related to the enzyme flexibility.

**Table 2:** Effect of temperature on kinase activity of PGK1 wild type and variants.

	T_max_ (°C)	*E*_a_ (kcal/mol)
Wild type	37	11.47 ± 0.7
R38M	40	9.44 ± 0.34
R65W	37	10.03 ± 0.60
G166D	37	6.00 ± 0.30
M189I	37	8.50 ± 0.35
A199V	37	9.11 ± 0.39
V216F	40	12.82 ± 0.70
F241S	35	6.39 ± 0.41

*E*_a_ was determined by the Arrhenius equation Eq (1) in the temperature range between 10°C and the optimal temperature of each protein.

### Thermal stability

The thermal stability of PGK1 wild type and variants was studied between 20°C and 80°C by continuously monitoring the ellipticity changes at 222 nm, where the main amplitude was observed (S2 Fig). The thermal denaturation process is irreversible, as indicated by the spectra measured at the end of the cooling phase that differ from those of the native proteins measured at the beginning of the thermal transitions. The temperature-induced ellipticity changes at 222 nm occur in an apparent cooperative transition and overlap with the increase of the photomultiplier tube voltage above 370 V (data not shown) at increasing temperature, suggesting that the temperature-induced unfolding is accompanied by protein aggregation, as revealed by the presence of a large amount of precipitate in the cuvette at the end of the cooling phase. The parameter chosen to compare the transition curves of the proteins is the melting temperature (T_m_) defined as the midpoint of the denaturation process and calculated by plotting the first derivative of the molar ellipticity values as a function of temperature (S2 Fig, inset). T_m_ values range from 49.0°C to 53.5°C being 52.5°C the T_m_ obtained for the wild type protein (Table 3). The variant R65W shows the same T_m_ value of the wild type, a modest increase in T_m_ value is observed for the variants G166D and A199V; all the other variants show T_m_ values lower than that of the wild type, with F241S showing a T_m_ value three degrees below that of the wild type (Table 3). Notably, differences in the amplitude observed for the thermal transitions of most of the variants (S2 Fig) may be referred to the difference in the dichroic activity at 222 nm of their corresponding native states, as also indicated in far-UV CD spectra reported in S1 Fig.

**Table 3:** Melting temperatures and thermodynamic parameters for urea-induced unfolding equilibrium of PGK1 wild type and variants measured by far-UV CD and fluorescence spectroscopy.

	T_m_ (°C)	$\Delta G^{H_{2}O}$ (kcal/mol)		*m* (kcal/mol•M)		[Urea]_0.5_ (M)	
		CD ([Θ]222)	Fluorescence $\left(\bar{\lambda }\right)$	CD ([Θ]222)	Fluorescence $\left(\bar{\lambda }\right)$	CD ([Θ]222)	Fluorescence $\left(\bar{\lambda }\right)$
Wild type	52.5	8.29 ± 0.57	8.06 ± 0.46	3.54 ± 0.25	3.10 ± 0.17	2.34	2.60
R38M	51.5	6.91 ± 0.73	5.54 ± 0.37	3.54 ± 0.37	1.87 ± 0.12	1.95	2.96
R65W	52.5	7.04 ± 0.32	7.20 ± 0.66	3.07 ± 0.14	2.63 ± 0.24	2.29	2.73
G166D	53.0	4.19 ± 0.31	4.94 ± 0.29	2.33 ± 0.16	1.51 ± 0.09	1.75	3.27
M189I	50.5	4.87 ± 0.31	5.01 ± 0.48	2.36 ± 0.15	1.82 ± 0.17	2.06	2.75
A199V	53.5	7.14 ± 0.57	6.82 ± 0.72	3.41 ± 0.14	2.71 ± 0.22	2.23	2.63
V216F	52.0	6.45 ± 0.34	5.76 ± 0.54	2.99 ± 0.16	2.14 ± 0.20	2.16	2.69
F241S	49.0	3.02 ± 0.17	3.80 ± 0.26	2.06 ± 0.10	1.67 ± 0.11	1.47	2.27

T_m_ values were calculated by taking the first derivative of the ellipticity at 222 nm with respect to temperature. Urea-induced unfolding equilibrium data were measured as described in Materials and Methods by monitoring the ellipticity at 222 nm ([Θ_222_]) and fluorescence intensity averaged emission wavelength $\left(\bar{\lambda }\right)$. $\Delta G^{H_{2}O}$ and *m* values were obtained from Eq (3); [Urea]_0.5_ was calculated from Eq (5). Data are reported as the mean ± SE of the fit.

### Urea-induced equilibrium unfolding transitions

PGK1 wild type and variants reversibly unfold in urea at 20°C in 20 mM Tris-HCl pH 8.0, 1 mM EDTA, 200 mM NaCl and 0. 2 mM DTT. The effect of increasing urea concentrations (0–8 M) on the protein structure was analyzed by far-UV CD and fluorescence spectroscopy (S3 Fig). The fluorescence changes at increasing urea concentration were measured by calculating the intensity averaged emission wavelength $\bar{\lambda }$, an integral measurement that depends both on the position and the shape of the spectrum. The same samples used to monitor the fluorescence changes during the unfolding transitions were used to monitor the far-UV CD ellipticity changes, to allow a direct comparison of the data. The urea-induced changes in 222 nm ellipticity and in the emission fluorescence show a sigmoidal dependence on urea concentration and follow an apparent two-state transition without any detectable intermediate (S3 Fig). The unfolding process is fully reversible upon dilution of the denaturant either for the wild type and the variant proteins. The denaturation curves relative to the apparent two-state equilibrium unfolding measured by far-UV CD and by fluorescence have been fitted to a two-state model according to Eq (4) to obtain the thermodynamic parameters $\Delta G^{H_{2}O}$, the free energy change for unfolding in the absence of denaturant, and *m*, a parameter that refers to the amount of protein surface area exposed to the solvent during unfolding (Table 3) [46]. Interestingly, the *m* values determined either for PGK1 wild type and variants are lower than that predicted for a protein of 417 amino acid residues unfolded in urea [47, 48], suggesting a more complex, presumably non-two state, unfolding process with undetectable intermediates (S3 Fig). $\Delta G^{H_{2}O}$ values of PGK1 variants are similar to those of the wild type, with the exception of G166D, M189I and F241S that show a significant decrease in the thermodynamic parameters (Table 3), mainly due to a decrease in *m* values. A decrease in *m* value may suggest either a destabilization of the native state or the presence of unfolding intermediates, not detectable at the equilibrium. The occurrence of undetectable unfolding intermediates may be the case of R38M and G166D, as suggested by the non coincidence of the [Urea]_0.5_ values obtained by far-UV CD and fluorescence changes, mainly due to the differences in *m* value (Table 3).

### Structural analysis

We investigated the structure of PGK1 variants R38M, G166D, V216F and M189I in the presence of MgADP and 3-PG through X-ray crystallography in order to reveal possible local and global variations with respect to the wild type protein. Two different crystallographic conformations of PGK1 have been already identified: the partially closed conformation, able to bind substrates, and the closed conformation, the catalytically competent state. Another form, defined as open and that is the most populated conformation in solution, has been detected through small-angle X-ray scattering [7]. It is interesting to note that all the mutants crystallized in the partially closed conformation but only M189I crystallized even in the closed form, suggesting that for the variants R38M, G166D and V216F the closed form is less favourable in the crystallization conditions. The overall structure is conserved in all the mutants compared to the wild type (Table 4, S4 Fig). The details of local variation are discussed case-by-case.

**Table 4:** Superimposition between the PGK1 wild type and the crystallized variants.

Mutant	Domain	Role/position of mutated residue	Conformation	Ligands	RMSD^#^ (Å^2^)
R38M	3-PG-binding	Binding of 3-PG, charge balancing in the transition state	Partially closed	MgADP	vs 2ZGV*: 0.23 vs 2XE7*: 0.96
G166D	3-PG-binding	Close to 3-PG COO^-^	Partially closed	MgADP	vs 2ZGV*: 0.62 vs 2XE7*: 1.17
V216F	ADP-binding	Part of the catalytic loop 211–219 that favors closed conformation	Partially closed	MgADP, 3-PG	vs 2ZGV*: 0.60 vs 2XE7*: 1.22
M189I	Hinge	N-terminal portion of the hinge	Partially closed	MgADP, 3-PG	vs 2ZGV*: 0.60 vs 2XE7*: 1.18
			Closed	MgADP, 3-PG	vs 2WZB*: 0.25

RMSD^#^: root mean square deviation obtained from the superimposition of each variant with respect to wild type PGK1. 2ZGV*: wild type binding MgADP; 2XE7*: wild type binding MgADP and 3-PG; 2WZB*: wild type binding MgADP, 3-PG and MgF_3_^-^

#### R38M variant

**Fig 3:**
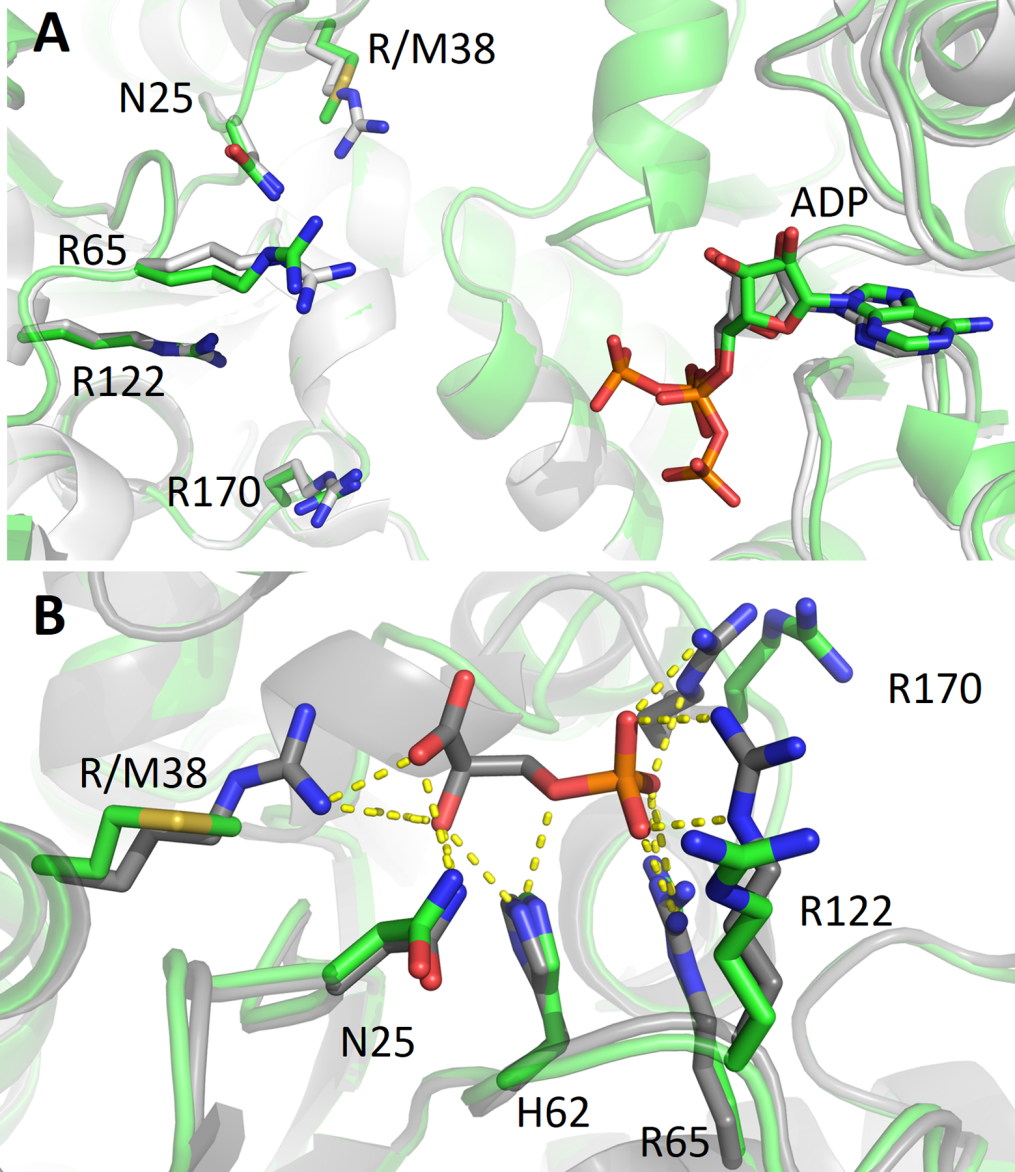
Detail of the 3-PG binding site in the variant R38M in comparison with PGK1 wild type. Overlap of R38M variant (green) and PGK1 wild type crystallized (A) in the absence of 3-PG (PDB 2ZGV) (light grey) or (B) in the presence of 3-PG (PDB: 2XE7) (grey). The view in B is rotated about 90° anticlockwise with respect to A. ADP, 3-PG and the residues involved in 3-PG binding are shown as sticks.

The residue R38 is placed in the N-terminal 3-PG binding domain and is a residue important for the substrate binding and its correct positioning towards the co-substrate MgADP (Fig 3). Moreover, together with residues K215 and K219, R38 is critical for the charge balancing of the transition state, directly interacting with the transferring phosphate group in the closed conformation of PGK1 [10].

The crystal structure of R38M contains ADP but does not contain 3-PG (Fig 3A), in line with the role of R38. The absence of the 3-PG ligand could account even for the variant reluctance to crystallize in the closed form since the binding of both substrates is required for the stabilization of this conformation. Taken together these observations justify the dramatic effect of R38M mutation on kinetic parameters (see Table 1). Indeed, upon R38M mutation the *K*_M_ increases from 0.40 to 3.15 mM and the turnover number strongly decreases from 89.8 to 7.2x10^-6^ s^-1^.

Apart from the mutation, in position 38, the local geometry of residues belonging to the 3-PG binding pocket is very similar to that of the wild type protein crystallized in the absence of 3-PG (PDB code: 2ZGV, Fig 3B). The only differences between the two structures concern the α-helix 374–382, visible in the R38M variant but not in the wild type 2ZGV structure, and the position of the β-phosphate group, which in R38M points towards this helix (Fig 3A). However, both the helix and the phosphate present some variability upon different PGK1 structures indicating that it could be dependent on crystallization conditions.

#### G166D variant

**Fig 4:**
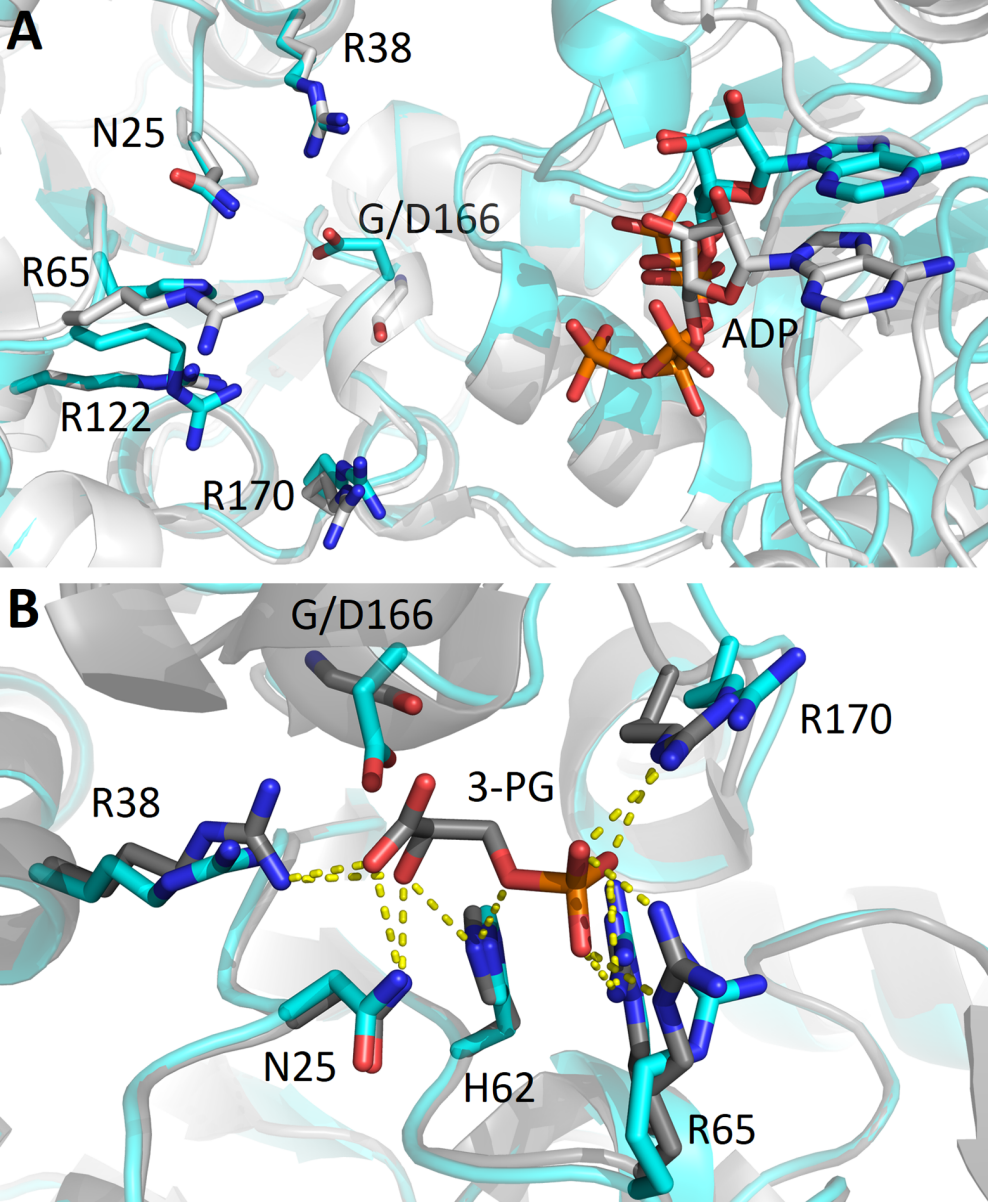
Detail of the 3-PG binding site in the variant G166D in comparison with PGK1 wild type. Overlap of G166D variant (cyan) and PGK1 wild type crystallized (A) in the absence of 3-PG (PDB 2ZGV) (light grey) or (B) in the presence of 3-PG (PDB: 2XE7) (grey). The view in B is rotated about 90° anticlockwise with respect to A.

G166 is one of the residues lining the 3-PG binding site and its substitution for the bulky and negatively charged aspartate modifies the shape and the charge of the substrate binding pocket. The structure of G166D (Fig 4) reveals that the aspartate Oδ is engaged in a salt bridge with the R38 NH1 (OD-(D166)-NH1(Arg38) = 3.3 Å) altering its capability of interacting with 3-PG. These modifications of the pocket impair the binding of 3-PG, that is not present in the structure, and the stabilization of the transition state through R38, thus affecting both *k*_cat_ (5.9 s^-1^) with a 15-fold decrease, and *K*_M_ (1.54 mM) increased 4- fold.

#### V216F variant

**Fig 5:**
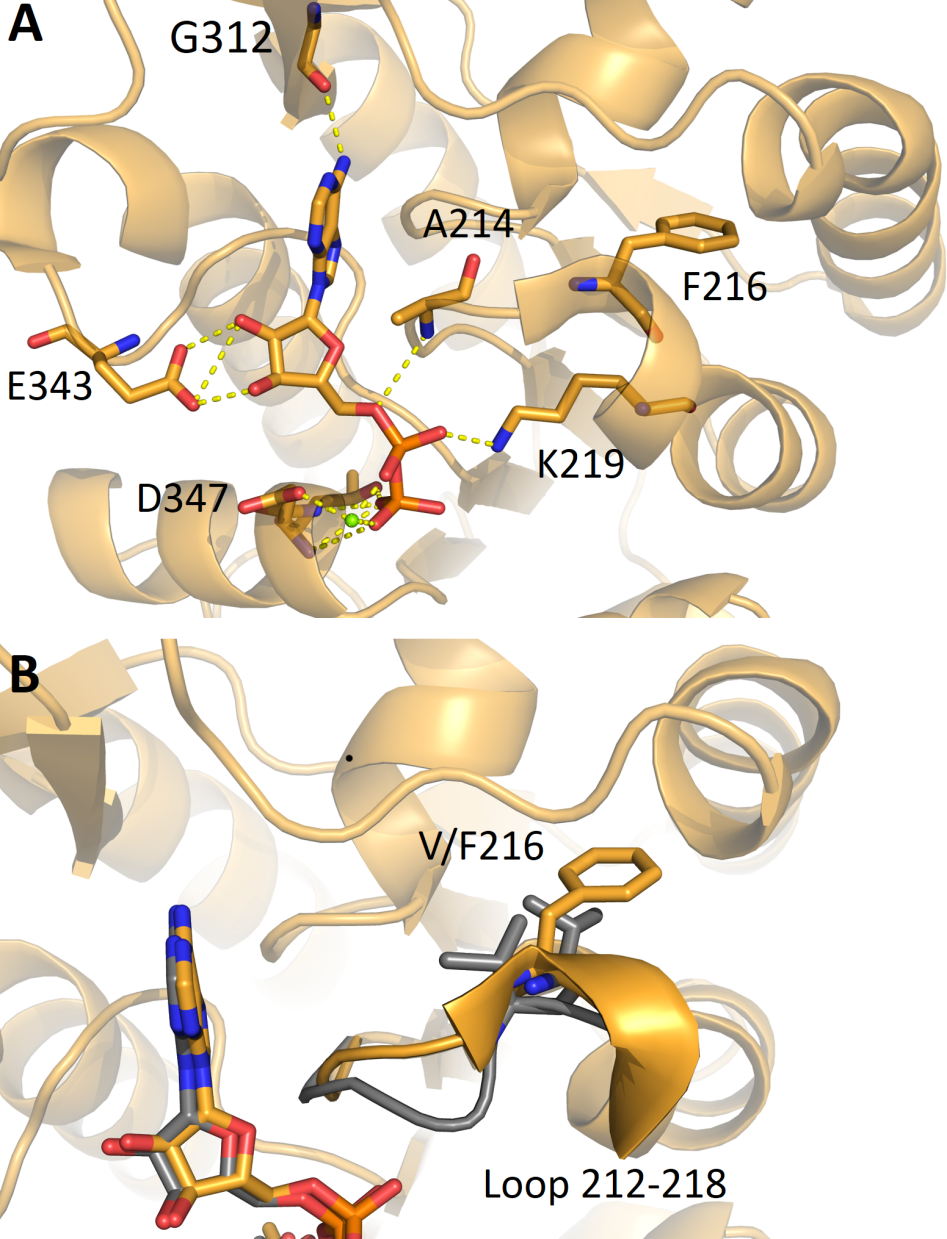
ADP binding site in the variant V216F in comparison with PGK1 wild type. (A) Detail of the binding of ADP to V216F variant (orange). ADP and the residues involved in the interaction are depicted as sticks, Mg^2+^ ion as a green sphere. (B) Superimpostition with wild type PGK1 crystallized with ADP and 3-PG (PDB: 2XE7) (grey), highlighting how the mutation V216F impairs the movement of the loop 212–218. The residue V/F216 and ADP are shown as sticks.

V216 belongs to the loop 214–219, close to the ADP-binding site. The rearrangement of this loop has been suggested to be important for the enzyme transition to the closed conformation upon binding of the two ligands [7]. Indeed, in the wild type structure in complex with the MgADP ligand the loop assumes two conformations, promoting the reorientation of K215 and D218, now ready to interact with γ-phosphate of 3-PG and D65, in the closed conformation. In the V216F variant structure in complex with MgADP, the loop adopts the same conformation as in apo-PGK1. As shown in Fig 5B, replacement of the valine with a phenylalanine keeps the loop in the apo-like conformation since the aromatic residue tends to stay buried. The lower mobility of the loop hampers the transition from the open to the closed conformation affecting the enzyme catalytic activity (the *k*_cat_ of the mutant is 1.9 s^-1^, 45-fold lower than that of the wild type). Conversely, no variation is found in ADP-binding. In fact, as shown in Fig 5, the residues directly involved in the ADP binding (E343, G312, A214) and the Mg^2+^ion bound through the β and γ-phosphate and D374 preserve the same positions as in the wild type structure.

#### M189I variant

**Fig 6:**
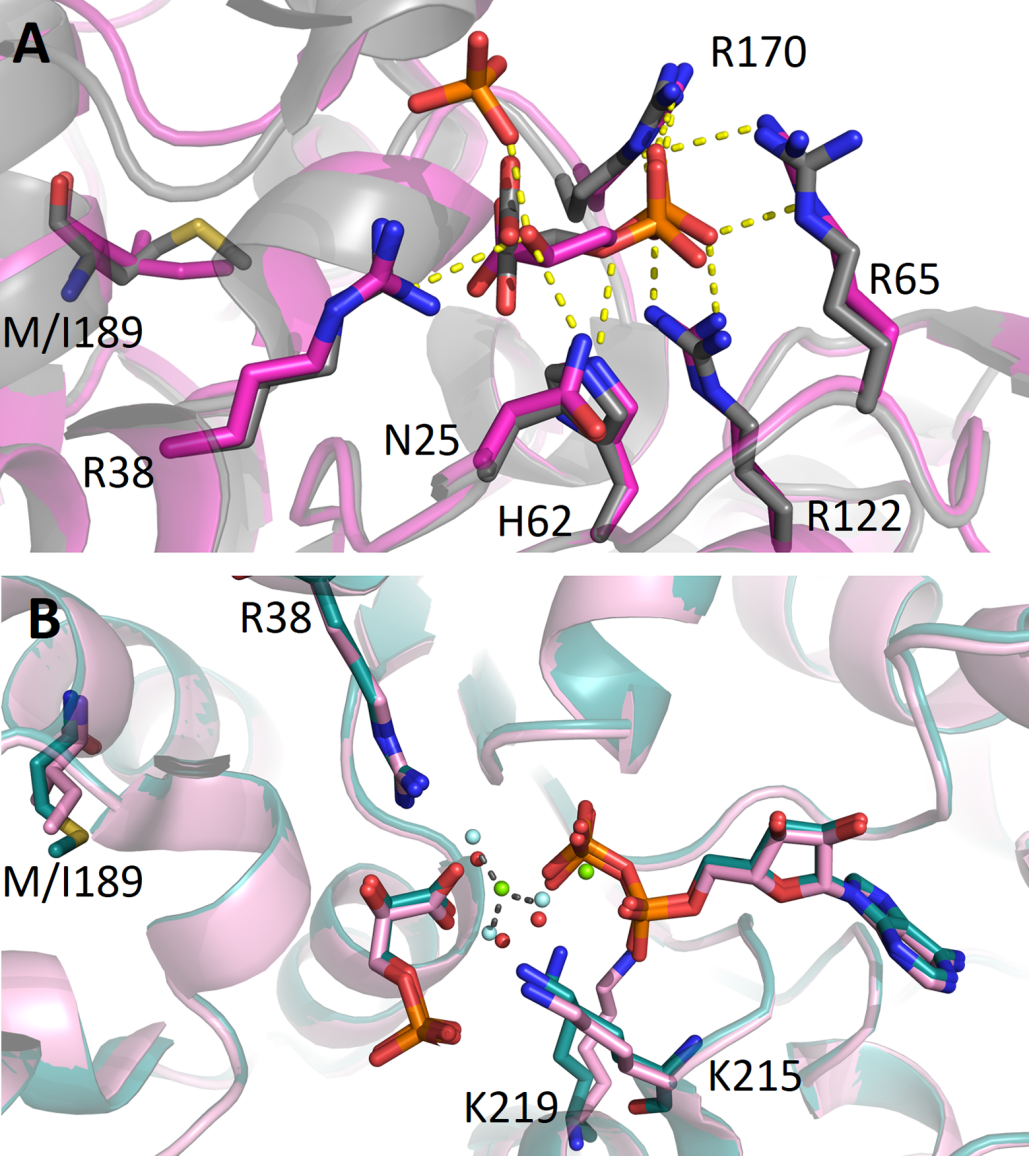
M189I variant in comparison with PGK1 wild type. (A) Detail of the binding of 3-PG to M189I variant in the partially-closed conformation (magenta) superimposed with partially-closed wild type PGK1 (PDB: 2XE7) (grey). 3-PG and the residues involved in the interaction are depicted as sticks. (B) Superimpostition of M189I variant in the closed conformation (pink) with wild type PGK1 crystallized with ADP, 3-PG and MgF_3_^-^ (PDB: 2WBZ) (deep teal). MgF_3_^-^ is shown as green and cyan spheres. Three water molecules (red spheres) bound to M189I mimic the position of F^-^ in MgF_3_^-^.

Residue 189 is part of the 189–201 α-helix, the structural element that represents the hinge connecting the N-terminal 3-PG binding domain and the C-terminal ADP binding domain. The structure of M189I has been solved both in open and closed conformation. Similarly to the other variants, M189I partially open structure is coincident to that of the wild type but in this case both ADP and 3-PG are clearly visible. It should be noticed that in the M189I variant structure the hydroxyl carboxylate moiety of the 3-PG is 90°-rotated with respect to the position adopted in the wild type structure. This could be due to the presence, in the active site, of a phosphate ion which may induce a change in the mode of 3-PG binding (Fig 6A). Since M189I mutation causes neither overall nor local conformational change, the *K*_M_ of the mutant is very similar to that of the wild type protein (0.58 mM *vs* 0.40 mM) and the *k*_cat_ is only three times lower than that of the wild type protein (30.5 *vs* 89.8 s^-1^). The structure in the closed conformation was obtained in the same condition of the wild type protein (PDB code: 2WZB). As shown in Fig 6B, the structure appear to be identical to the wild type ones; also the position of the two ligands, ADP and 3-PG, is conserved. Interestingly, in the M189I structure in the closed conformation MgF_3_^-^ is not present, therefore the transition complex, evident in the wild type structure, is not formed in the M189I variant. Nevertheless, all the residues stabilizing the transition state are superposable between the wild type and mutant structure.

## Discussion

Phosphoglycerate kinase 1 (PGK1) is an ubiquitous enzyme expressed in all somatic cells that plays a central role in glycolysis where it provides energy in form of ATP through the reversible phosphotransfer reaction from 1,3-bisphosphoglycerate (1,3-BPG) to MgADP in order to produce 3-phosphoglycerate (3-PG) and MgATP in the presence of free magnesium. PGK1 is a central enzyme in the cancer cells metabolism since they utilize preferentially glycolysis to produce ATP. Alteration of PGK1 levels has been reported in different cancer types and this enzyme is considered as a negative prognostic marker [23]. In the general metabolic reprogramming of cancer cells, glycolytic enzymes play a pivotal role since many intermediates of the glycolytic pathway may also be utilized for the increased biosynthetic demand of proliferating tumor cells [49]. Indeed, one of the consequence of the metabolic change in cancer cells is the request of reducing equivalent for biosynthesis that can be provided through the deviation of glycolysis intermediate in the shunt of pentose phosphate pathway [50].

In this study, we selected from the COSMIC database some somatic PGK1 single nucleotide variants found in cancer tissues. The variants involve amino acid residues located in the enzyme 3-PG binding domain, in the nucleotide binding domain and in the hinge region connecting the two domains (Fig 1) and are different from those associated to PGK1 deficiency and hereditary non-spherocytic haemolytic anaemia [33].

Independently from the position of the mutation on the protein, the main result of the aminoacid substitutions, object of this report, is a significant change in the variants catalytic properties. All the variants display a reduced catalytic efficiency, particularly evident for the variant R38M, that shows a decrease of eight order of magnitude of the *k*_cat_/K_M_ value, mainly due to a dramatic decrease of the turnover number (Table 1). As shown in the structure (Fig 3), R38 is located in the 3-PG binding site and interacts electrostatically with the carboxyl group of 3-PG, hence it is critical for the positioning of the substrate in the catalytic site and for the stabilization of the transition state (Fig 6B). Accordingly, the substitution of the R38 implies a dramatic decrease of the *k*_cat_ and an increase of *K*_m_ (see Table 1).

Mutation of the other residues involved in 3-PG binding, R65W, a positively charged arginine with the non polar tryptophan, and G166D, a substitution of glycine with a negatively charged residue, leads to an increase in *K*_m_ values and, in the case of G166D, also to a reduced turnover number. As shown in the structure, the introduction of a negatively charged residue in the 3-PG binding site alters the affinity of the protein for the substrate (which is not found in the structure) by occupying part of the binding site and by engaging the R38 in a new salt bridge (Fig 4). Notably, the binding affinity for 3-PG is also significantly reduced in the variant A199V, a mutation placed at the end of the hinge region. Regarding the mutants of the nucleotide binding domain, V216F shows a decrease in the turnover number of about fifty fold, while F241S a notable increase in the *K*_m_ value accompanied by a two-fold increase in the turnover number. Interestingly, the turnover numbers of M189I and A199V, both in the hinge region and that of R65W, in the 3-PG binding site, are comparable to the turnover number of the wild type, indicating that these mutations are not affecting the release of the product. The reduced catalytic efficiency of all variants is supported by the local structural changes observed in the X-ray structure of the crystallized variants that involve mutation of residues located either in the 3-PG binding domain (R38M, G166D) or in the nucleotide binding site (V216F) or in the hinge (M189I). Notably, the structural comparative analysis shows that the introduced mutations do not cause large conformational changes in the protein tridimensional structure but only local changes involving the mutated residues and their chemical surrounding. These local changes may be responsible for local differences in protein flexibility that become evident in the values of activation energy that is decreased for all the cancer associated variants, with the notable exception of V216F. In this variant, the introduction of a phenylalanine residue reinforces the hydrophobic interactions between the 211–219 loop and the 243–263 region thereby stabilizing the C-terminal domain (Fig 5). This local stabilization, which decreases the flexibility of the ATP binding site, is sufficient to determine the decrease of about 50-fold of the turnover number with respect to that of the wild type protein. The global effects of local alterations are evident from the analysis of the thermodynamic parameters that point to a destabilization of the native state, as indicated by the decrease of $\Delta G^{H_{2}O}$ values for all the variants, particularly evident for G166D, M189I and F241S that involve mutation of residues located in the 3-PG binding site, in the hinge region and in the nucleotide binding site, respectively (Fig 1). Indeed, the significant decrease of the *m* values observed for G166D, M189I and F241S suggests a decrease of their surface area exposed to the solvent during unfolding.

The reduced catalytic properties and the decrease of the stability of PGK1 variants may suggest that their expression in the neoplastic cells would significantly burden the metabolic flux through the glycolytic pathway. Thus, metabolic intermediates may accumulate and be converted in other products through alternative pathway, such as the pentose shunt, that could supply for the biosynthetic need of the growing and proliferating tumor cell. On the other hand, the occurrence of a “glycolytic jam” may induce the cell to increase the uptake of glucose [50].

Glycolytic enzymes may play additional roles other than sustaining the cell metabolism, and translocate from the cytoplasm into the nucleus and/or mitochondria thus displaying additional non-metabolic functions [51, 52]. Thus, mutations in a glycolytic enzyme may affect not only its metabolic activity but also its alternative non-metabolic function. PGK1, as a moonlighting enzyme, play alternative roles in different cellular compartments. In the mitochondria, PGK1 acts as a protein kinase and inhibits pyruvate metabolism promoting the Warburg effect [14]. In the nucleus, PGK1 binds to DNA polymerases α and εand increases the synthesis of DNA [13]. In addition PGK1, when secreted in extracellular compartment by tumor cells, binds plasmin thus permitting the cleavage of plasminogen in order to generate the vascular inhibitor angiostatin [53].

It is noteworthy that some of the PGK1 variants found in cancer tissues involve residues important for mitochondrial translocation, i.e. the region between residues 38 and 43. In this regard, R38, replaced with a methionine in the variant R38M, is placed in a critical position. Similarly, A199V is in close proximity to S203 which, prior to mitochondria translocation, is phosphorylated under hypoxia conditions [14].

In conclusion, the present paper discloses the structure and functional features of nsSNVs of PGK1 found in cancer cells: R38M, R65W, G166D, M189I, A199V, V216F and F241S. We succeed in solving the X-ray crystal structures of the variants with mutation in the N-terminal region (R38M and G166D), in the C-terminal region (V216F) and in the hinge region (M189I). We solved the structures of all these mutants in the partially closed conformation, and we succeed to solve the structure of M189I also in the totally closed conformation.

Our study reveals that most of the PGK1 nsSNVs found in cancer tissues display a decreased catalytic efficiency together with a destabilization of the native state. However, the structural and functional data show only local changes around the mutated residues, without large conformational changes in the protein structure. All these variants are less efficient with respect to the wild type protein and the loss of efficiency is related, as shown by the crystals structures, to the decreased affinity for the substrate as in the case of the R38M and G166D, or to an increased local rigidity of the substrates binding sites as in the case of V216F and M189I. A less efficient PGK1 in cancer cells may point to its moonlighting functions to satisfy cancer cell proliferation and survival requirements.

## Supporting information

S1 Table List of oligonucleotides used for site-directed mutagenesis (PDF). (PDF)

S2 Table Crystallization conditions, data collection parameters, refinement statistics of the PGK1 variants (PDF). (PDF)

S1 Fig Spectral properties of PGK1 wild type and variants (PDF). (PDF)

S2 Fig Thermal unfolding of PGK1 wild type and variants (PDF). (PDF)

S3 Fig Urea-induced equilibrium unfolding of PGK1 wild type and variants (PDF). (PDF)

S4 Fig Overall fold of PGK1 versions compared to the wild type (PDF). (PDF)

## References

[1] Siegel RL, Miller KD, Jemal A. Cancer statistics, 2016. CA Cancer J Clin. 2016;66:7–30. doi: 10.3322/caac.21332 26742998 10.3322/caac.21332

[2] Bertout JA, Patel SA, Simon MC. The impact of O_2_ availability on human cancer. Nat Rev Cancer. 2008;8: 967–975. doi: 10.1038/nrc2540 18987634 10.1038/nrc2540 PMC3140692

[3] Kroemer G, Pouyssegur J. Tumor cell metabolism: cancer’s Achilles’heel. Cancer Cell.2008;13:472–482. doi: 10.1016/j.ccr.2008.05.005 18538731 10.1016/j.ccr.2008.05.005

[4] Sheng H, Tang W. Glycolysis inhibitors for anticancer therapy: a review of recent patents. Recent Pat Anticancer Drug Discov. 2016;11:297–308. 27087655 10.2174/1574892811666160415160104

[5] Rodríguez-Enríquez S, Gallardo-Pérez JC, Hernández-Reséndiz I, Marín-Hernández A, Pacheco-Velázquez SC, López-Ramírez SY, et al Canonical and new generation anticancer drugs also target energy metabolism. Arch Toxicol. 2014;88:1327–1350. doi: 10.1007/s00204-014-1246-2 24792321 10.1007/s00204-014-1246-2

[6] Vas M, Varga A, Gráczer E. Insight into the mechanism of domain movements and their role in enzyme function: example of 3-phosphoglycerate kinase. Curr Protein Pept Sci. 2010;11:118–147. 20088776 10.2174/138920310790848403

[7] Zerrad L, Merli A, Schröder GF, Varga A, Gráczer É, Pernot P, et al A spring-loaded release mechanism regulates domain movement and catalysis in phosphoglycerate kinase. J Biol Chem. 2011;286:14040–14048. doi: 10.1074/jbc.M110.206813 21349853 10.1074/jbc.M110.206813 PMC3077604

[8] Beutler E. PGK deficiency. Br J Haematol. 2007;136:3–11. doi: 10.1111/j.1365-2141.2006.06351.x 17222195 10.1111/j.1365-2141.2006.06351.x

[9] Gondeau C, Chaloin L, Lallemand P, Roy B, Périgaud C, Barman T, et al Molecular basis for the lack of enantioselectivity of human 3-phosphoglycerate kinase. Nucleic Acids Res. 2008;36:3620–3629. doi: 10.1093/nar/gkn212 18463139 10.1093/nar/gkn212 PMC2441801

[10] Cliff MJ, Bowler MW, Varga A, Marston JP, Szabó J, Hounslow AM, et al Transition state analogue structures of human phosphoglycerate kinase establish the importance of charge balance in catalysis. J Am Chem Soc. 2010;132:6507–6516. doi: 10.1021/ja100974t 20397725 10.1021/ja100974t

[11] Chen X, Zhao C, Li X, Wang T, Li Y, Cao C, et al, Terazosin activates Pgk1 and Hsp90 to promote stress resistance. Nat Chem Biol. 2015;11:19–25. doi: 10.1038/nchembio.1657 25383758 10.1038/nchembio.1657 PMC4412158

[12] Wang J, Wang J, Dai J, Jung Y, Wei CL, Wang Y, et al A glycolytic mechanism regulating an angiogenic switch in prostate cancer. Cancer Res. 2007;67:149–159. doi: 10.1158/0008-5472.CAN-06-2971 17210694 10.1158/0008-5472.CAN-06-2971

[13] Boukouris AE, Zervopoulos SD, Michelakis ED. Metabolic Enzymes Moonlighting in the Nucleus: metabolic Regulation of Gene Transcription. Trends Biochem Sci. 2016;41:712–730. doi: 10.1016/j.tibs.2016.05.013 27345518 10.1016/j.tibs.2016.05.013

[14] Li X, Jiang Y, Meisenhelder J, Yang W, Hawke DH, Zheng Y, et al Mitochondria-translocated pgk1 functions as a protein kinase to coordinate glycolysis and the tca cycle in tumorigenesis. Mol Cell. 2016;61:705–719. doi: 10.1016/j.molcel.2016.02.009 26942675 10.1016/j.molcel.2016.02.009 PMC4888784

[15] Ariosa AR, Klionsky DJ. A novel role for a glycolytic pathway kinase in regulating autophagy has implications in cancer therapy. Autophagy. 2017;13: 1091–1092. doi: 10.1080/15548627.2017.1321723 28537472 10.1080/15548627.2017.1321723 PMC5529076

[16] Qian X, Li X, Cai Q, Zhang C, Yu Q, Jiang Y et al Phosphoglycerate Kinase 1 Phosphorylates Beclin1 to Induce Autophagy. Mol Cell. 2017 3;65:917–931. doi: 10.1016/j.molcel.2017.01.027 28238651 10.1016/j.molcel.2017.01.027 PMC5389741

[17] Ahmad SS, Glatzle J, Bajaeifer K, Buhler S, Lehmann T, Konigsrainer I, et al Phosphoglycerate kinase 1 as a promoter of metastasis in colon cancer. Int J Oncol. 2013;43: 586–590. doi: 10.3892/ijo.2013.1971 23727790 10.3892/ijo.2013.1971

[18] Ai J, Huang H, Lv X, Tang Z, Chen M, Chen T, et al FLNA and PGK1 are two potential markers for progression in hepatocellular carcinoma. Cell Physiol Biochem. 2011;27: 207–216. doi: 10.1159/000327946 21471709 10.1159/000327946

[19] Daly EB, Wind T, Jiang XM, Sun L, Hogg, PJ. Secretion of phosphoglycerate kinase from tumour cells is controlled by oxygen-sensing hydroxylases. Biochim Biophys Acta. 2004;1691:17–22. doi: 10.1016/j.bbamcr.2003.11.004 15053920 10.1016/j.bbamcr.2003.11.004

[20] Duan Z, Lamendola DE, Yusuf RZ, Penson RT, Preffer FI, Seiden MV. Overexpression of human phosphoglycerate kinase 1(PGK1) induces a multidrug resistance phenotype. Anticancer Res. 2002;22:1933–1941. 12174867

[21] Zieker D, Konigsrainer I, Tritschler I, Loffler M, Beckert S, Traub F, et al Phosphoglycerate kinase 1 a promoting enzyme for peritoneal dissemination in gastric cancer. Int J Cancer. 2010;126:1513–20. doi: 10.1002/ijc.24835 19688824 10.1002/ijc.24835 PMC2811232

[22] Hwang TL, Liang Y, Chien KY, Yu JS. Overexpression and elevated serum levels of phosphoglycerate kinase 1 in pancreatic ductal adenocarcinoma. Proteomics. 2006;6:2259–2272. doi: 10.1002/pmic.200500345 16493704 10.1002/pmic.200500345

[23] Sun S, Liang X, Zhang X, Liu T, Shi Q, Song Y, et al Phosphoglycerate kinase-1 is a predictor of poor survival and a novel prognostic biomarker of chemoresistance to paclitaxel treatment in breast cancer. J Cancer. 2015;112:1332–1339. 10.1038/bjc.2015.114 PMC4402453 25867275

[24] Forbes SA, Beare D, Boutselakis H, Bamford S, Bindal N, Tate J, et al COSMIC: somatic cancer genetics at high-resolution. Nucleic Acids Res. 2017;45(D1):D777–D783. doi: 10.1093/nar/gkw1121 27899578 10.1093/nar/gkw1121 PMC5210583

[25] Karki R, Pandya D, Elston RC, Ferlini C. Defining "mutation" and "polymorphism" in the era of personal genomics. BMC Med Genomics. 2015;8:37–43. doi: 10.1186/s12920-015-0115-z 26173390 10.1186/s12920-015-0115-z PMC4502642

[26] Pasquo A, Consalvi V, Knapp S, Alfano I, Ardini M, Stefanini S, et al Structural stability of human protein tyrosine phosphatase rho catalytic domain: effect of point mutations. PLoS One. 2012;7(2):e32555 doi: 10.1371/journal.pone.0032555 22389709 10.1371/journal.pone.0032555 PMC3289658

[27] Lori C, Lantella A, Pasquo A, Alexander LT, Knapp S, Chiaraluce R, et al Effect of single amino acid substitution observed in cancer on Pim-1 kinase thermodynamic stability and structure. PLoS One. 2013;8(6):e64824 doi: 10.1371/journal.pone.0064824 23755147 10.1371/journal.pone.0064824 PMC3673989

[28] Lori L, Pasquo A, Lori C, Petrosino M, Chiaraluce R, Tallant C, et al Effect of BET missense mutations on bromodomain function, inhibitor binding and stability. PLoS One. 2016;11(7):e0159180 doi: 10.1371/journal.pone.0159180 eCollection 2016. 27403962 10.1371/journal.pone.0159180 PMC4942050

[29] Petrosino M, Lori L, Pasquo A, Lori C, Consalvi V, Minicozzi V, et al, Single-Nucleotide Polymorphism of PPARγ, a Protein at the Crossroads of Physiological and Pathological Processes. Int J Mol Sci. 2017; 18(2). pii: E361 doi: 10.3390/ijms18020361 28208577 10.3390/ijms18020361 PMC5343896

[30] Casadio R, Vassura M, Tiwari S, Fariselli P, Martelli LP. Correlating disease-related mutations to their effect on protein stability: a large-scale analysis of the human proteome. Hum Mutat. 2011; 32:1161–1170. doi: 10.1002/humu.21555 21853506 10.1002/humu.21555

[31] Petukh M, Kucukkal TG, Alexov E. On human disease-causing amino acid variants: statistical study of sequence and structural patterns. Hum Mutat. 2015;36:524–534. doi: 10.1002/humu.22770 25689729 10.1002/humu.22770 PMC4409542

[32] Kucukkal TG, Petukh M, Li L, Alexov E. Structural and physico-chemical effects of disease and non-disease nsSNPs on proteins. Curr Opin Struct Biol. 2015;32:18–24. doi: 10.1016/j.sbi.2015.01.003 25658850 10.1016/j.sbi.2015.01.003 PMC4511717

[33] Chiarelli LR, Morera SM, Bianchi P, Fermo E, Zanella A, Galizzi A, et al Molecular insights on pathogenic effects of mutations causing phosphoglycerate kinase deficiency. PLoS One 2012;7(2):e32065 doi: 10.1371/journal.pone.0032065 22348148 10.1371/journal.pone.0032065 PMC3279470

[34] Gill SC, von Hippel PH. Calculation of protein extinction coefficients from amino acid sequence data. Anal Biochem. 1989;182:319–326. 2610349 10.1016/0003-2697(89)90602-7

[35] Benjwal S, Verma S, Rohm KH, Gursky O. Monitoring protein aggregation during thermal unfolding in circular dichroism experiments. Protein Sci. 2006;15:635–639. doi: 10.1110/ps.051917406 16452626 10.1110/ps.051917406 PMC2249783

[36] Royer CA, Mann CJ, Matthews CR. Resolution of the fluorescence equilibrium unfolding profile of trp aporepressor using single tryptophan mutants. Protein Sci. 1993;2:1844–1852. doi: 10.1002/pro.5560021106 8268795 10.1002/pro.5560021106 PMC2142281

[37] Santoro MM, Bolen DW. Unfolding free energy changes determined by the linear extrapolation method. 1. Unfolding of phenylmethanesulfonyl alpha-chymotrypsin using different denaturants. Biochemistry. 1988; 27:8063–8068. 3233195 10.1021/bi00421a014

[38] Kabsch W. XDS. Acta Crystallogr D Biol Crystallogr. 2010;D66:125–132. 10.1107/S0907444909047337 PMC2815665 20124692

[39] Evans PR and Murshudov GN. How good are my data and what is the resolution? Acta Cryst. 2013;D69, 1204–1214. 10.1107/S0907444913000061 PMC3689523 23793146

[40] Vagin A, Teplyakov A. MOLREP: an automated program for molecular replacement. J. Appl. Cryst. 1997; 30: 1022–1025.

[41] Murshudov GN, Vagin AA, Dodson EJ. Refinement of macromolecular structures by the maximum-likelihood method. Acta Crystallogr D Biol Crystallogr. 1997;53(Pt 3):240–255. doi: 10.1107/S0907444996012255 15299926 10.1107/S0907444996012255

[42] Emsley P, Cowtan K. Coot: model-building tools for molecular graphics. Acta Crystallogr D Biol Crystallogr. 2004;60(Pt 12 Pt 1):2126–2132. 15572765 10.1107/S0907444904019158

[43] Sherman MA, Fairbrother WJ, Mas MT. Characterization of the structure and properties of the His62fAla and Arg38fAla mutants of yeast phosphoglycerate kinase: an investigation of the catalytic and activatory sites by site-directed mutagenesis and NMR. Prot Sci. 1992;1:752–760. 10.1002/pro.5560010607 PMC2142244 1304916

[44] Choy N, Raussens V, Narayanaswami V. Inter-molecular coiled-coil formation in human apolipoprotein E C-terminal domain. J Mol Biol. 2003;334:527–539. 14623192 10.1016/j.jmb.2003.09.059

[45] Kiss RS, Weers PM, Narayanaswami V, Cohen J, Kay CM, Ryan RO. Structure-guided protein engineering modulates helix bundle exchangeable apolipoprotein properties. J Biol Chem. 2003;278:21952–21959. doi: 10.1074/jbc.M302676200 12684504 10.1074/jbc.M302676200

[46] Myers JK, Pace CN, Scholtz JM. Denaturant m values and heat capacity changes: Relation to changes in accessible surface areas of protein unfolding. Protein Sci. 1995; 4:2138–2148. doi: 10.1002/pro.5560041020 8535251 10.1002/pro.5560041020 PMC2142997

[47] Auton M, Holthauzen LM, Bolen DW. Anatomy of energetic changes accompanying urea-induced protein denaturation. Proc Natl Acad Sci USA. 2007; 104:15317–15322. doi: 10.1073/pnas.0706251104 17878304 10.1073/pnas.0706251104 PMC2000523

[48] Geierhaas CD, Nickson AA, Lindorff-Larsen K, Clarke J, Vendruscolo M. BPPred: A computational tool to predict biophysical quantities of proteins. Protein Sci. 2007;16:125–134. doi: 10.1110/ps.062383807 17123959 10.1110/ps.062383807 PMC2222837

[49] Pavlova NN, Thompson CB. The emerging hallmarks of cancer metabolism. Cell Metab. 2016; 23:27–47. doi: 10.1016/j.cmet.2015.12.006 26771115 10.1016/j.cmet.2015.12.006 PMC4715268

[50] Lim SO, Li CW, Xia W, Lee HH, Chang SS, Shen J, et al EGFR signaling enhances aerobic glycolysis in triple negative breast cancer cells to promote tumor growth and immune escape Cancer Res. 2016;76:1284–1296. doi: 10.1158/0008-5472.CAN-15-2478 26759242 10.1158/0008-5472.CAN-15-2478 PMC4775355

[51] Yu X, Li S. Non-metabolic functions of glycolytic enzymes in tumorigenesis. Oncogene. 2017;36: 2629–2636. doi: 10.1038/onc.2016.410 27797379 10.1038/onc.2016.410

[52] Lu Z, Hunter T. Metabolic Kinases Moonlighting as Protein Kinases. Trends Biochem Sci. 2018; pii: S0968-0004(18)30020-3. doi: 10.1016/j.tibs.2018.01.006 [Epub ahead of print] 29463470 10.1016/j.tibs.2018.01.006 PMC5879014

[53] Butera D, Wind T, Lay AJ, Beck J, Castellino FJ, Hogg PJ. Characterization of a reduced form of plasma plasminogen as the precursor for angiostatin formation. J Biol Chem. 2014; 289:2992–3000. doi: 10.1074/jbc.M113.539924 24338014 10.1074/jbc.M113.539924 PMC3908430

