# Supplementary material for: The phosphoglycerate kinase 1 variants found in carcinoma cells display different catalytic activity and conformational stability compared to the native enzyme
Source: PLoS One. 2018 Jul 11;13(7):e0199191. doi: 10.1371/journal.pone.0199191 (PMC6040698; doi:10.1371/journal.pone.0199191)
Supplement: S1 Table — (PDF) [file pone.0199191.s001.pdf]

Supporting Information

S1 Table. List of oligonucleotides used for site-directed mutagenesis

Mutant	Primer sequences (5' to 3')
R38M	**FW**ACAACCAG**ATG**ATTAAGGCTGCTGT<br>**REV**ACAGCAGCCTTAAT**CAT**CTGGTTGT
R65W	**FW** CACCTAGGC**TGG**CCTGATGGTGT<br>**REV** ACACCATCAGG**CCA**GCCTAGGTG
G166D	**FW** GATGCTTTT**GAC**ACTGCTCACAG<br>**REV** CTGTGAGCAGT**GTC**AAAAGCATC
M189I	**FW** GCTGGTGGGTTTTTG**ATC**AAGAAGGAGCTG<br>**REV** CAGCTCCTTCTT**GAT**CAAAAACCCACCAGC
A199V	**FW** C**TTT**GCAAAG**GTC**TTGGAGAGCCCA**G**<br>**REV** CTGGGCTCTCCAA**GAC**CTTTGCAAAG
V216F	**FW** CGGAGCTAAA**TTT**GCAGACAAGATC<br>**REV**GATCTTGTCTGC**AAA**TTTAGCTCCG
F241S	**FW** GGAATGGCT**TCT**ACCTTCCTTAAGG<br>**REV** CCTTAAGGAAGGT**AGA**AGCCATTCC
