# Supplementary material for: The phosphoglycerate kinase 1 variants found in carcinoma cells display different catalytic activity and conformational stability compared to the native enzyme
Source: PLoS One. 2018 Jul 11;13(7):e0199191. doi: 10.1371/journal.pone.0199191 (PMC6040698; doi:10.1371/journal.pone.0199191)
Supplement: S2 Table — (PDF) [file pone.0199191.s002.pdf]

S2 Table. Crystallization conditions, data collection parameters, refinement statistics of the PGK1 variants.

	**V216F**	**R38M**	**G166D**	**M189I** (partially closed)	**M189I** (closed)
PDB codes	5M3U	5O7D	5M1R	5M6Z	5MXM
Resolution (Å)	1.81	1.84	1.64	1.67	2.05
Crystallization conditions	2.0 M Na/KPO_4, pH 8.4	Na citrate 1.6 M pH 6.5	Na citrate 1.6 M pH 6.5	2.5 M Na/KPO_4, pH 8.6	PEG 2K MME 30% w/v, bis-tris 0.1 M pH 7
X-ray source	ESRF-BM30-A3	ESRF ID23-2	ESRF ID23-2	ESRF ID23-2	BESSY 14.3
Wavelength (Å)	0.9677	0.8729	0.8729	0.8729	0.8943
Space group	$P2_1$	$P2_1$	$P2_1$	$P2_1$	$P2_12_12_1$
Unit cell parameters *a,b,c* (Å); β (°)	35.65, 106.03, 50.10; 98.71	36.05, 106.68, 50.69; 97.32	35.76, 105.57, 50.00; 97.76	35.88, 106.12, 50.47; 98.13	38.36, 91.10, 109.10; 90
No. of molecules in the asymmetric unit (modelled residue range)	1 (1-416)	1 (2-416)	1 (1-416)	1 (1-416)	1 (2-416)
Wilson B-factor ($Å^2$)	26.3	17.4	17.1	22.4	18.3
Data analysis range (highest resolution shell) (Å)	1.81-50 (1.81-1.92)	1.84–50 (1.84-1.87)	1.64-52.78 (1.64-1.74)	1.67-50 (1.67-1.77)	2.05-69.91 (2.05-2.17)
Unique reflections	32377	31685	44562	42601	24685
Completeness (%)	96 (79.1)	96.1 (99.5)	98.7 (97.3)	97.5 (94.1)	99.7 (99.3)
Redundancy	4.5 (3.8)	6.9 (6.9)	3.7 (3.7)	4.2 (4.2)	6.0 (5.9)
^{a}Rmerge	0.08 (0.56)	0.17 (0.89)	0.097 (57)	0.054 (0.67)	0.14 (0.70)
CC(1/2) (%)	99.7 (75.3)	99.6 (36.7)	99.3 (68.8)	99 (59)	99.5 (88.3)
$\langle I/\sigma(I)\rangle$	11.22 (1.88)	10.5 (2.2)	7.84 (1.82)	15.08 (1.89)	13.15 (3.13)
Refinement ranges (highest resolution bin)	1.81-50 (1.81-1.85)	1.84-53.34 (1.84-1.89)	1.64-52.78 (1.64-1.66)	1.67-53.06 (1.67-1.71)	2.05-50 (2.05-2.10)
R_{crys} (%)	19.4 (29.3)	22.8 (36.3)	16.6 (25.9)	18.5 (30.1)	17.9 (29.5)
R_{free} (%)	24.2 (38.6)	26.7 (36.6)	20.1 (30.5)	22.3 (33.2)	22.6 (39.5)
rms (angles) (°)	0.015	0.011	0.012	0.011	0.016
rms (bonds) (Å)	1.735	1.209	1.667	1.558	1.95
Residues in allowed region of Ramachandran plot / generously allowed (%)	100 / 2	100 / 3	100 / 2	100 / 1	100 / 2

Values in parentheses are for the highest-resolution shell.

$^a R_{merge} = \sum_{hkl}\sum_i |I_i(hkl) - \langle I(hkl)\rangle| / \sum_{hkl}\sum_i I_i(hkl)$, where $I_i(hkl)$ is the ith observation of the reflection (hkl) and $\langle I(hkl)\rangle$ is the mean intensity of the (hkl) reflection.

S1 Fig

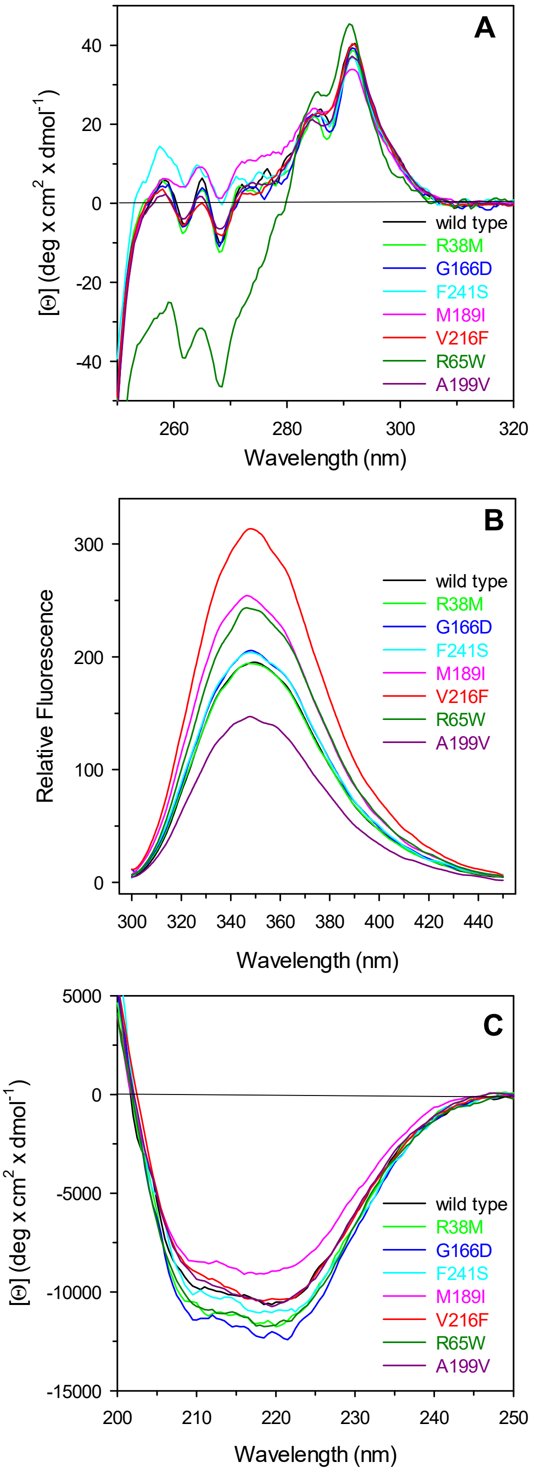

S1 Fig. Spectral properties of PGK1 wild type and variants. (A) Near-UV CD spectra were recorded in a 1.0-cm quartz cuvette at 1.5-1.7 mg/mL protein concentration in 20 mM Tris-HCl pH 8.0 containing 2.0 mM DTT, 1 mM EDTA and 200 mM NaCl. (B) Intrinsic fluorescence emission spectra were recorded at 110 µg/mL for R65W and 130 µg/mL for wild type and the other variants (0.08 AU_{280nm}, 295 nm excitation wavelength), in 20 mM Tris-HCl, pH 8.0 containing 0.2 M NaCl and 0.2 mM DTT. (C) Far-UV CD spectra were recorded in a 0.1-cm quartz cuvette at 130-170 µg/mL in 20 mM Tris-HCl, pH 7.5 containing 0.2 M NaCl and 0.2 mM DTT. All spectra were recorded at 20°C.
