# Supplementary material for: The phosphoglycerate kinase 1 variants found in carcinoma cells display different catalytic activity and conformational stability compared to the native enzyme
Source: PLoS One. 2018 Jul 11;13(7):e0199191. doi: 10.1371/journal.pone.0199191 (PMC6040698; doi:10.1371/journal.pone.0199191)
Supplement: S1 Fig — (PDF) [file pone.0199191.s003.pdf]

S1 Fig

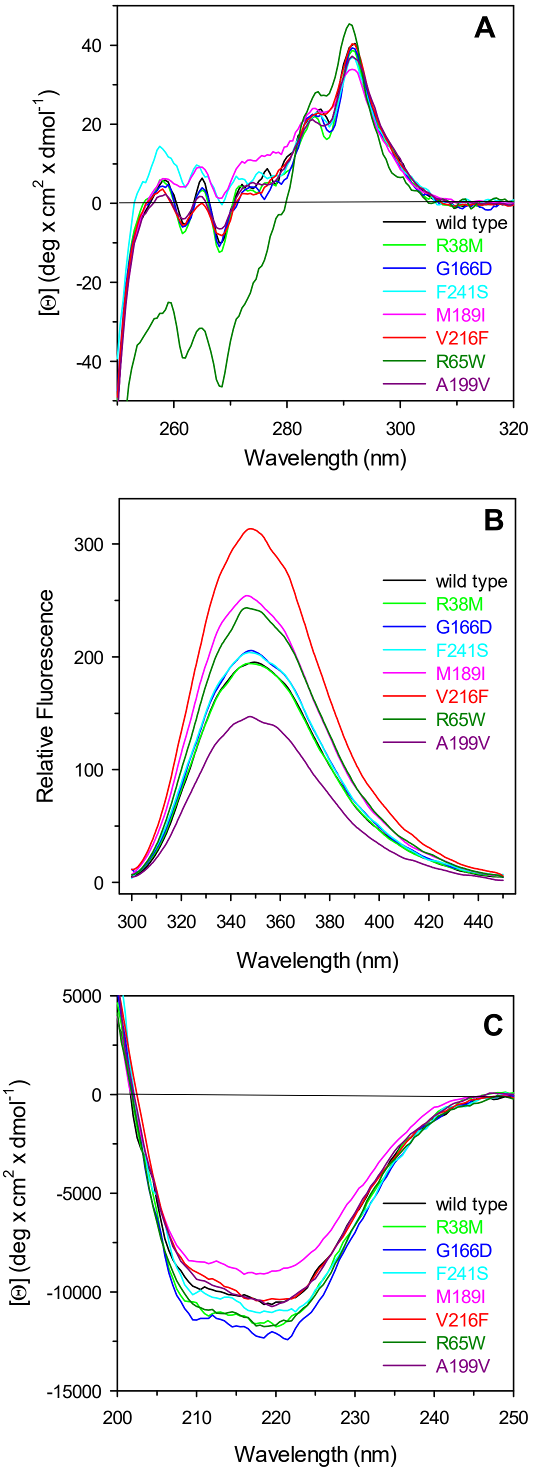

S1 Fig. Spectral properties of PGK1 wild type and variants. (A) Near-UV CD spectra were recorded in a 1.0-cm quartz cuvette at 1.5-1.7 mg/mL protein concentration in 20 mM Tris-HCl pH 8.0 containing 2.0 mM DTT, 1 mM EDTA and 200 mM NaCl. (B) Intrinsic fluorescence emission spectra were recorded at 110 µg/mL for R65W and 130 µg/mL for wild type and the other variants (0.08 AU_{280nm}, 295 nm excitation wavelength), in 20 mM Tris-HCl, pH 8.0 containing 0.2 M NaCl and 0.2 mM DTT. (C) Far-UV CD spectra were recorded in a 0.1-cm quartz cuvette at 130-170 µg/mL in 20 mM Tris-HCl, pH 7.5 containing 0.2 M NaCl and 0.2 mM DTT. All spectra were recorded at 20°C.
