# Supplementary material for: The phosphoglycerate kinase 1 variants found in carcinoma cells display different catalytic activity and conformational stability compared to the native enzyme
Source: PLoS One. 2018 Jul 11;13(7):e0199191. doi: 10.1371/journal.pone.0199191 (PMC6040698; doi:10.1371/journal.pone.0199191)
Supplement: S2 Fig — (PDF) [file pone.0199191.s004.pdf]

S2 Fig

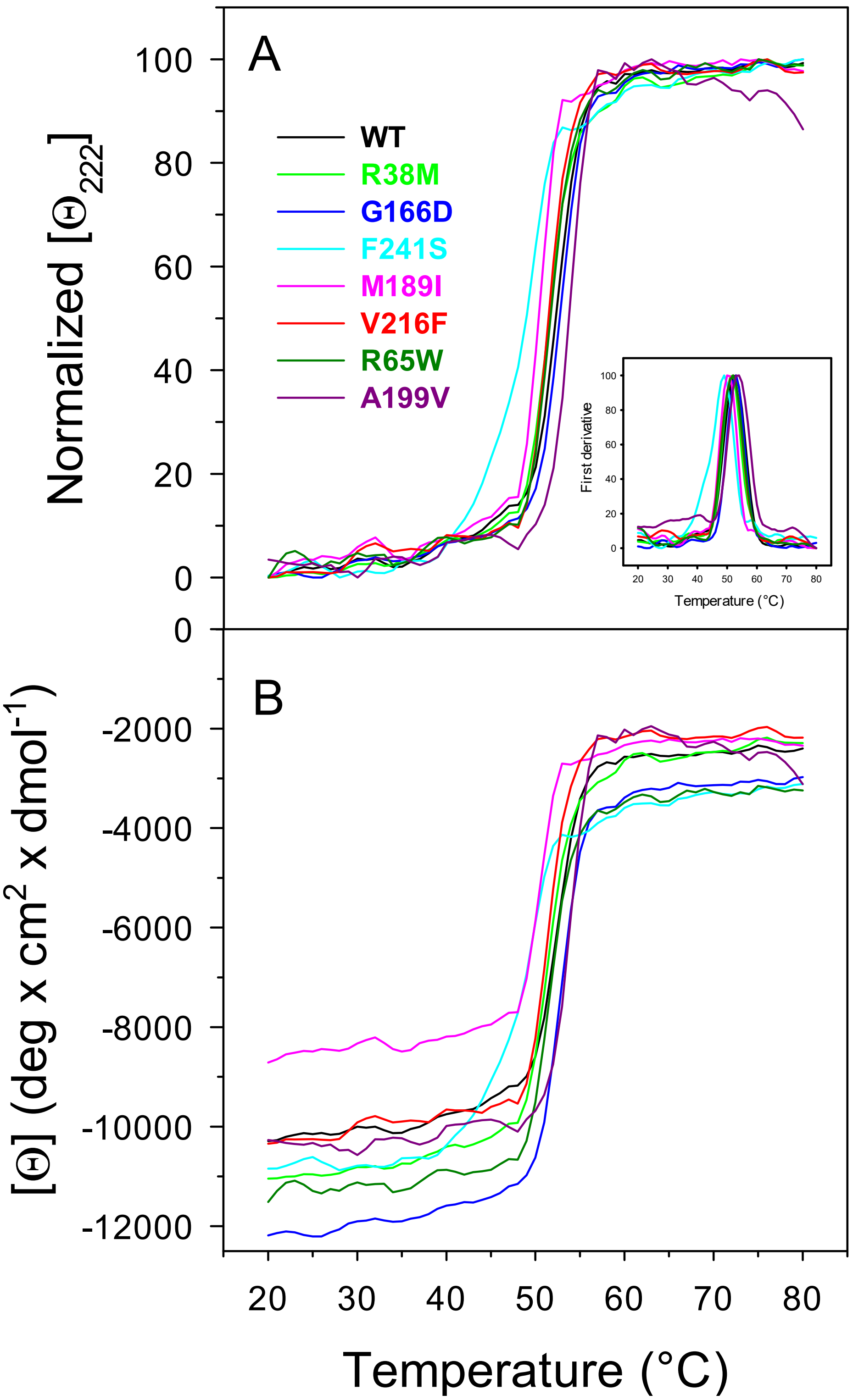

S2 Fig. Thermal unfolding of PGK1 wild type and variants. PGK1 variants and wild type (130-170 μg/mL) were heated from 20°C to 80°C in 20 mM Tris-HCl, pH 8.0 containing 0.2 M NaCl and 0.2 mM DTT . The molar ellipticity at 222 nm ($[\Theta_{222}]$) was monitored continuously every 0.5 °C. (A) Normalized $[\Theta_{222}]$; the inset shows the first derivative of the same data as in (A). (B) $[\Theta_{222}]$ before normalization.
