# Supplementary material for: The phosphoglycerate kinase 1 variants found in carcinoma cells display different catalytic activity and conformational stability compared to the native enzyme
Source: PLoS One. 2018 Jul 11;13(7):e0199191. doi: 10.1371/journal.pone.0199191 (PMC6040698; doi:10.1371/journal.pone.0199191)
Supplement: S3 Fig — (PDF) [file pone.0199191.s005.pdf]

S3 Fig

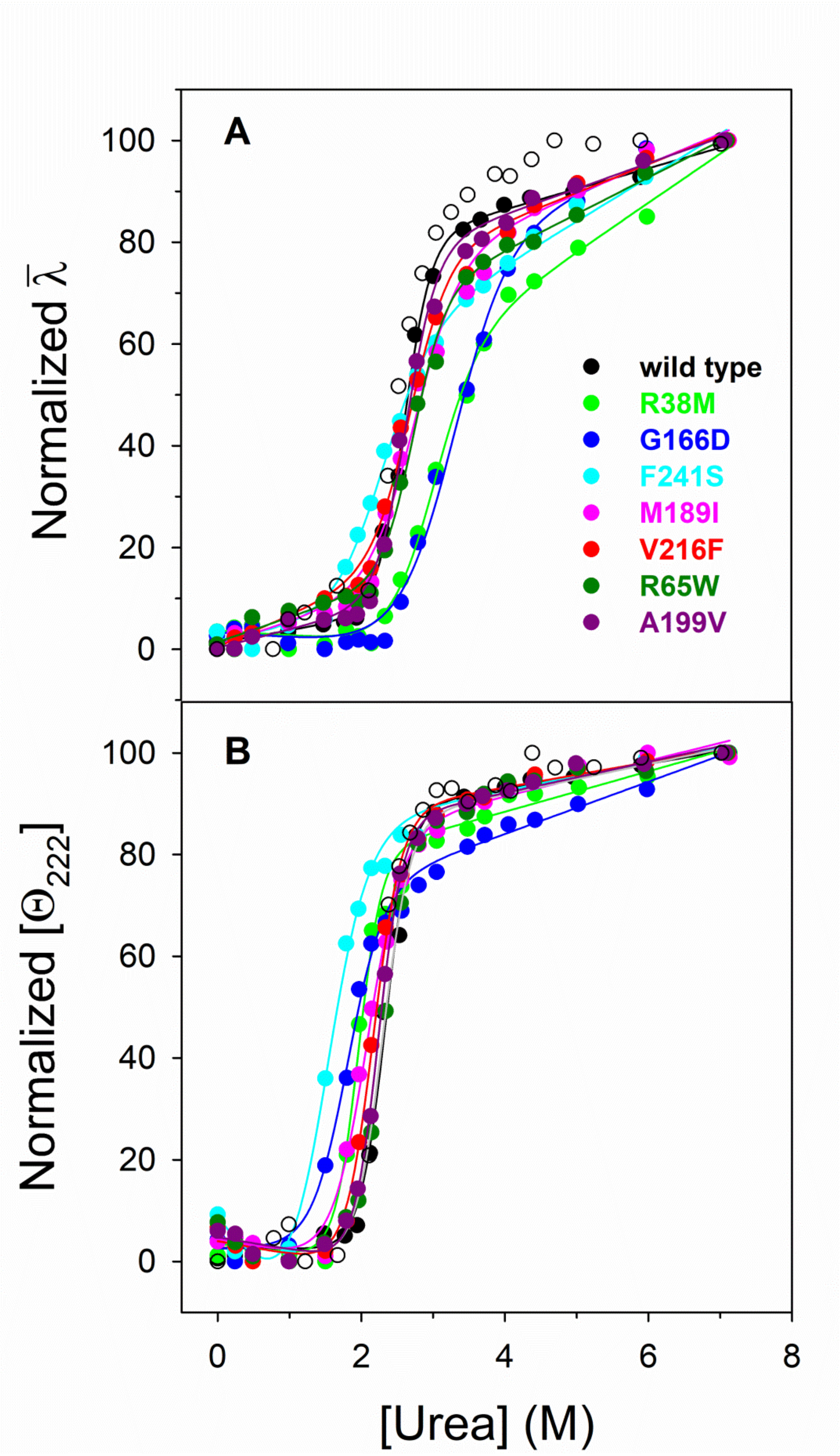

S3 Fig. Urea-induced equilibrium unfolding of PGK1 wild type and variants. (A) Normalized intensity-averaged emission wavelength ($\bar{\lambda}$). (B) Normalized molar ellipticity at 222 nm ([Θ_{222}]) reported after removal of the high-frequency noise and the low-frequency random error by SVD. The continuous lines represent the nonlinear fitting of the normalized $\bar{\lambda}$ and [Θ_{222}] data to Eq 4. The reversibility points (empty circles) are shown, for clarity, only for the wild type and were not included in the nonlinear regression analysis. All spectra were recorded at 20 °C, as described in Materials ad Methods.
