# Supplementary material for: The phosphoglycerate kinase 1 variants found in carcinoma cells display different catalytic activity and conformational stability compared to the native enzyme
Source: PLoS One. 2018 Jul 11;13(7):e0199191. doi: 10.1371/journal.pone.0199191 (PMC6040698; doi:10.1371/journal.pone.0199191)
Supplement: S4 Fig — (PDF) [file pone.0199191.s006.pdf]

S4 Fig

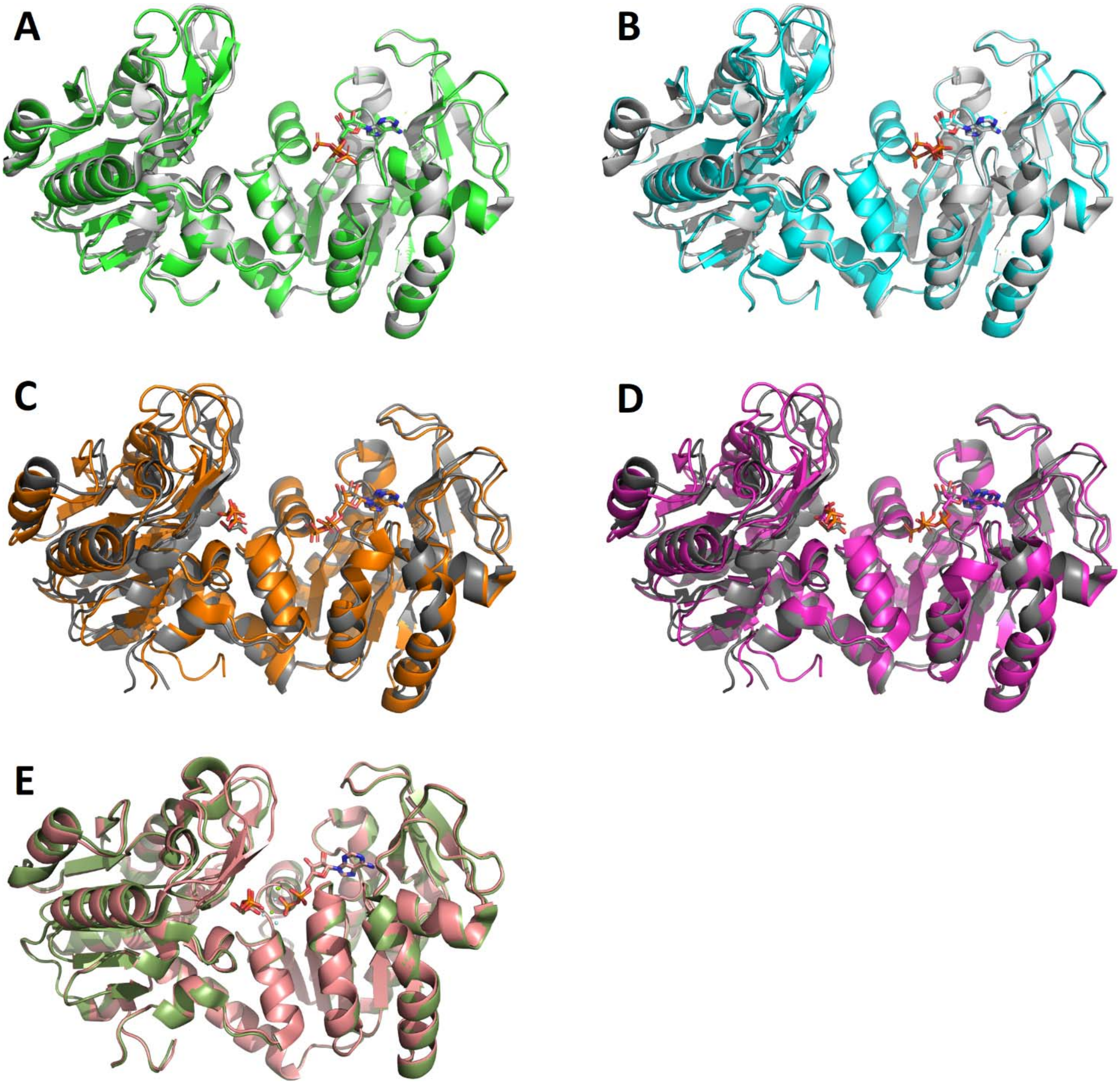

S4 Fig . Overall fold of PGK1 versions compared to the wild type. The structure of each variant has been superimposed with the most related structure of wild type, chosen according to the bound ligands and the conformation assumed. (A) R38M (green) *versus* 2ZGV (light grey). (B) G166D (cyan) versus 2ZGV (light grey). (C) V216F (orange) versus 2XE7 (dark grey). (D) M189I in partially open conformation (magenta) versus 2XE7 (dark grey). (E) M189I in closed conformation (pink) versus 2WZB (pale green). 2ZGV: wild type binding Mg-ADP; 2XE7: wild type binding Mg-ADP and 3-PG; 2WZB: wild type binding Mg-ADP, 3-PG and $MgF3^-$. Mg-ADP and 3-PG are shown as sticks, $MgF3^-$is shown as spheres.
